# Differentiation, Quantification and Identification of Abrin and *Abrus precatorius* Agglutinin

**DOI:** 10.3390/toxins13040284

**Published:** 2021-04-18

**Authors:** Sylvia Worbs, Bettina Kampa, Martin Skiba, Eva-Maria Hansbauer, Daniel Stern, Hervé Volland, François Becher, Stéphanie Simon, Martin B. Dorner, Brigitte G. Dorner

**Affiliations:** 1 Biological Toxins, Centre for Biological Threats and Special Pathogens, Robert Koch Institute, Seestr. 10, 13353 Berlin, Germany; worbss@rki.de (S.W.); kampab@rki.de (B.K.); skibam@rki.de (M.S.); eva.hansbauer@gmail.com (E.-M.H.); sternd@rki.de (D.S.); dornerm@rki.de (M.B.D.); 2 Département Médicaments et Technologies pour la Santé, Université Paris Saclay, CEA, INRAE, SPI, 91191 Gif-sur-Yvette, France; herve.volland@cea.fr (H.V.); francois.becher@cea.fr (F.B.); stephanie.simon@cea.fr (S.S.)

**Keywords:** Abrin, *Abrus precatorius*, monoclonal antibodies, ELISA, mass spectrometry, lateral flow assay, clinical sample, food, suicide attempt

## Abstract

Abrin, the toxic lectin from the rosary pea plant *Abrus precatorius,* has gained considerable interest in the recent past due to its potential malevolent use. However, reliable and easy-to-use assays for the detection and discrimination of abrin from related plant proteins such as *Abrus precatorius* agglutinin or the homologous toxin ricin from *Ricinus communis* are sparse. To address this gap, a panel of highly specific monoclonal antibodies was generated against abrin and the related *Abrus precatorius* agglutinin. These antibodies were used to establish two sandwich ELISAs to preferentially detect abrin or *A. precatorius* agglutinin (limit of detection 22 pg/mL for abrin; 35 pg/mL for *A. precatorius* agglutinin). Furthermore, an abrin-specific lateral flow assay was developed for rapid on-site detection (limit of detection ~1 ng/mL abrin). Assays were validated for complex food, environmental and clinical matrices illustrating broad applicability in different threat scenarios. Additionally, the antibodies turned out to be suitable for immuno-enrichment strategies in combination with mass spectrometry-based approaches for unambiguous identification. Finally, we were able to demonstrate for the first time how the developed assays can be applied to detect, identify and quantify abrin from a clinical sample derived from an attempted suicide case involving *A. precatorius*.

## 1. Introduction

The shrub *Abrus precatorius* belongs to the *Fabaceae* family and is also known as jequirity bean, crab’s eye, rosary or paternoster pea plant. It can be found in many tropical and subtropical areas across the globe. The seeds contain the highly toxic lectin abrin, a member of the type II ribosomal inactivating proteins (RIPs) family. These cytotoxic lectins facilitate cell death by halting protein synthesis by depurinating a specific adenine in the sarcin-loop of the ribosomal RNA. Type II RIPs consist of a catalytically active A-chain—the RNA N-glycosidase—and a sugar-binding B-chain—the lectin part—which mediates cellular binding and uptake [1]. A number of highly toxic plant proteins including ricin (castor oil plant, *Ricinus communis*) and viscumin (mistletoe, *Viscum album*) are members of the RIP family, which also includes shiga toxins produced by the bacteria *Escherichia coli* and *Shigella dysenteriae* [2,3,4]. Plant RIPs are thought to protect the producing organism against predators and fungi, whereas bacterial RIPs act as potent pathogenicity factors. Due to their high toxicity, RIPs are investigated in agriculture (pest control) or medicine (immunotoxins, anti-tumor and anti-viral activity) and some have also gained military or criminal interest [5,6,7,8]. Here, ricin is the best-known example, but abrin has also been used with malevolent intent recently; consequently, both toxins are listed as select agents [9,10]. Several reports cover the use of abrin in attempted murders or in a biothreat scenario [11,12,13,14], and accidental or suicidal poisonings with *Abrus precatorius* seeds have been reported [15]. Its lethality depends on the amount of seeds and the application route. Fatality after oral ingestion is usually rare in humans, while injection seems to be more severe [16,17,18,19,20,21,22,23]—a characteristic that has been noted for ricin previously [24]. Toxicological data from animal studies indicate that the major toxin abrin—as with other protein toxins—is more potent when applied systemically (half maximal lethal dose, LD_50_, in rats: 0.3–0.5 µg/kg) compared to inhalation (rat LD_50_ 3–4 µg/kg) and least toxic via the oral route (mice LD_50_ 2–3 mg/kg) [15,25]; however, the exact concentration and purity of the abrin used in those studies remain elusive. *Abrus precatorius* roots and leaves have been used in traditional (ayurvedic) medicine—e.g., to treat coughing—while the seeds can be laxative and abortive [7]. Moreover, boiled (detoxified) seeds can be part of the local diet in regions where *Abrus* is common [7]. Cooking or baking is used for other legumes (*Fabaceae*) as well, such as beans and lentils, to destroy their heat-labile toxic compounds, making them suitable for consumption.

*Abrus precatorius* seeds contain not only the toxic lectin abrin in four different isoforms (abrin-a, abrin-b, abrin-c, abrin-d), but also another highly related lectin named *A. precatorius* agglutinin (APA). While abrin is a canonical A-B toxin of 63–67 kDa, APA is a dimer (~ 134 kDa) of two non-covalently linked A’-B’ molecules of 67 kDa each. It is worthy of note that APA is far less toxic compared to abrin but acts as a hemagglutinin [26,27]; consequently, APA is not considered a select agent. APA’s and abrin’s subchains share >70% identity at amino acid level (Table 1), which makes discrimination between these two molecules challenging.

Apart from abrin and APA, the seeds also contain the *A. precatorius*-specific low molecular weight substance L-abrine (*N*-methyl-L-tryptophan; 218 Da). L-abrine has been used as a surrogate biomarker for *Abrus*-intoxications and can be assessed by mass spectrometry (MS) techniques [28]. However, L-abrine will be lost from preparations if abrin is further purified, e.g., by affinity chromatography [26,29,30].

While abrin and APA are both markers for *Abrus precatorius* intoxications, the discrimination between abrin and APA is fundamental in criminal or forensic investigations. Abrin is the major toxic compound and is classified as a select agent, with all the legal implications of this, whilst APA is of much lower toxicity and is not a select agent [10,26,27]. More importantly, the quantitative assessment of both abrin and APA is needed for the attribution of different *Abrus* preparations in the course of an investigation. In this context, the presence and quantity of APA provides information on any purification or refinement process that might have been applied.

Only a few immunological and MS-based assays have been described that directly detect the presence of the toxic compound abrin. Immunological assays such as sandwich enzyme-linked immunosorbent assays (ELISAs) can detect between 100 to 4000 pg/mL abrin and often work quite well with complex matrices such as beverages or foods [31,32,33,34]. Methods for on-site detection such as lateral flow assays (LFAs) can reach detection limits between 100 and 50,000 pg/mL but deliver results in less than an hour compared to the approximately four to six hours required for a conventional sandwich ELISA performed in microtiter plates [34,35,36,37]. LFAs are optimized for use by non-trained personnel in the field, and they are usually more prone to matrix effects due to the lack of washing steps. Although lab-based ELISAs are more time-consuming, they are cost-effective, can be automated and are applicable for high-throughput testing. Antibody and aptamer-based biosensors for abrin detection applying cantilevers, micro/nano optical fibres, Raman spectrometry or colorimetry have also been reported but to date have not been challenged against detection from complex matrices [38,39,40,41]. To assess the potential hazard in security and food safety scenarios, the discrimination of abrin from APA and other related toxins such as ricin is an important issue. Due to the high sequence homology between abrin and APA, only very few assays are able to distinguish between both molecules. The discrimination and unambiguous identification of abrin and APA has been achieved by MS-based methods delivering sequence information [42,43,44].

A basic prerequisite for any kind of method suitable for use in detection in complex matrices—which can be seen in different fields from clinical diagnosis to food safety and to criminal/forensic investigations—is the availability of highly specific and sensitive detection reagents. Antibodies—in particular, monoclonal antibodies (mAbs)—are still unmatched by other binding reagents. 

Here, we describe the generation and comprehensive characterization of a panel of mAbs against abrin and APA. The antibodies provided the basis to develop and validate ELISAs and LFAs suitable for the detection of abrin and APA from food, clinical and environmental samples. Additionally, selected mAbs turned out to be useful for immuno-enrichment strategies followed by MS-based identification and quantification. Finally, in a real case of attempted suicide by oral *A. precatorius* ingestion, the ELISA and MS methods were successfully applied to confirm abrin poisoning from fecal samples.

## 2. Results

### 2.1. Generation and Characterization of Monoclonal Antibodies Against Abrin and A. precatorius Agglutinin

As a starting point for the generation and characterization of antibodies against abrin and APA, three different lectin preparations were purified from *A. precatorius* seeds: first, a purified mixture of *A. precatorius* lectins devoid of non-carbohydrate binding proteins and low molecular weight metabolites containing abrin and APA in a ratio of approximately 2:3 as determined by Matrix-Assisted Laser Desorption Ionization–Time of Flight Mass Spectrometry (MALDI-TOF MS). Independently, a second preparation of highly pure abrin containing all four isolectins abrin-a, abrin-b, abrin-c, and abrin-d and a third preparation of highly pure APA were purified by chromatographic separation. Since abrin and APA have the same molecular weight under non-reducing conditions in an SDS-PAGE assay (Supplementary Figure S1), the quality control of the purified materials was performed by MALDI-TOF MS (Supplementary Figure S2). The purity of both the abrin and the APA preparation was estimated by mass spectrometry to be ≥97% [45]. While the mixture of *A. precatorius* lectins was used for the immunization of animals, the highly pure preparations of abrin and APA were used to select hybridoma clones and to characterize the binding profiles of the corresponding antibodies.

In order to generate monoclonal and polyclonal antibodies against abrin and APA, mice and a rabbit were immunized with the purified mixture of *A. precatorius* lectins containing abrin and APA. Considering the toxicity of abrin, the mixture of *A. precatorius* lectins was inactivated by formaldehyde treatment to generate a toxoid before administration as described in [46]. Once mice had mounted a substantial titer, they were boosted with the native *A. precatorius* lectin mixture to stimulate B-cells’ production of antibodies specific for the active toxin. Hybridoma cells producing mAbs were obtained after the fusion of splenocytes with myeloma cells [46,47]. 

In the first screening round, hybridoma supernatants were tested for their specificity to the mixture of *A. precatorius* lectins (abrin and APA) and the related proteins ricin and RCA120. While abrin and APA are 74.8% identical at amino acid level, abrin shares 48.8% sequence identity with ricin and 44.6% with RCA120, respectively (Table 1). Antibodies specific to the mixture of *A. precatorius* lectins and showing cross-reactivity against ricin and RCA120 were excluded [48]. In a second screening round, hybridoma supernatants were tested for their specific binding of abrin and/or APA using the above-mentioned highly pure preparations of abrin or APA, respectively. In total, about 5300 hybridoma supernatants from three individual fusions were tested for the production of specific antibodies. Fifteen positive hybridoma clones were selected and subcloned at least twice, and the corresponding antibodies were purified for further characterization. 

**Figure 1:**
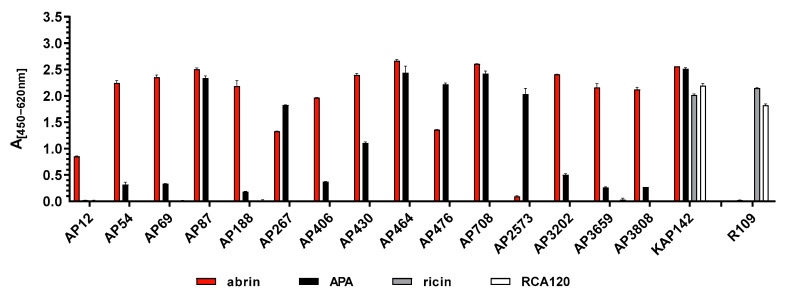
Specificity of monoclonal antibodies against abrin or APA in an indirect ELISA. Abrin (red), APA (black), ricin (grey) and RCA120 (white) were coated as antigens at 500 ng/mL each in 50 μL PBS containing 1 µg/mL BSA. The binding of the indicated monoclonal antibodies selected in this work (AP12 to AP3808 at 10 µg/mL) to the coated antigens was tested. For comparison and as a positive control, the polyclonal antibody KAP142 was used in parallel. As a negative control for abrin and APA, the monoclonal antibody R109 [46] was applied which specifically binds to ricin and RCA120 but does not detect the *Abrus* lectins.

To determine antibody specificity, the 15 mAbs and the polyclonal rabbit antibody (KAP142) were tested for binding to abrin, APA, ricin and *Ricinus communis* agglutinin (RCA120) by ELISA (Figure 1) and/or Western blotting (Supplementary Figure S3). As shown in Figure 1, three out of 15 monoclonal antibodies recognized abrin and APA equally well (AP87, AP464, and AP708) indicating specific binding to an epitope shared between abrin and APA. While nine monoclonal antibodies preferentially detected abrin (AP12, AP54, AP69, AP188, AP406, AP430, AP3202, AP3659, and AP3808) and three preferentially detected APA (strong preference: AP2573; weak preference: AP267 and AP476), none of the mAbs showed cross-reactivity towards ricin or RCA120 (Figure 1). In contrast, the polyclonal antibody KAP142 turned out to be reactive against both abrin and APA and was cross-reactive against ricin and RCA120.

**Figure 2:**
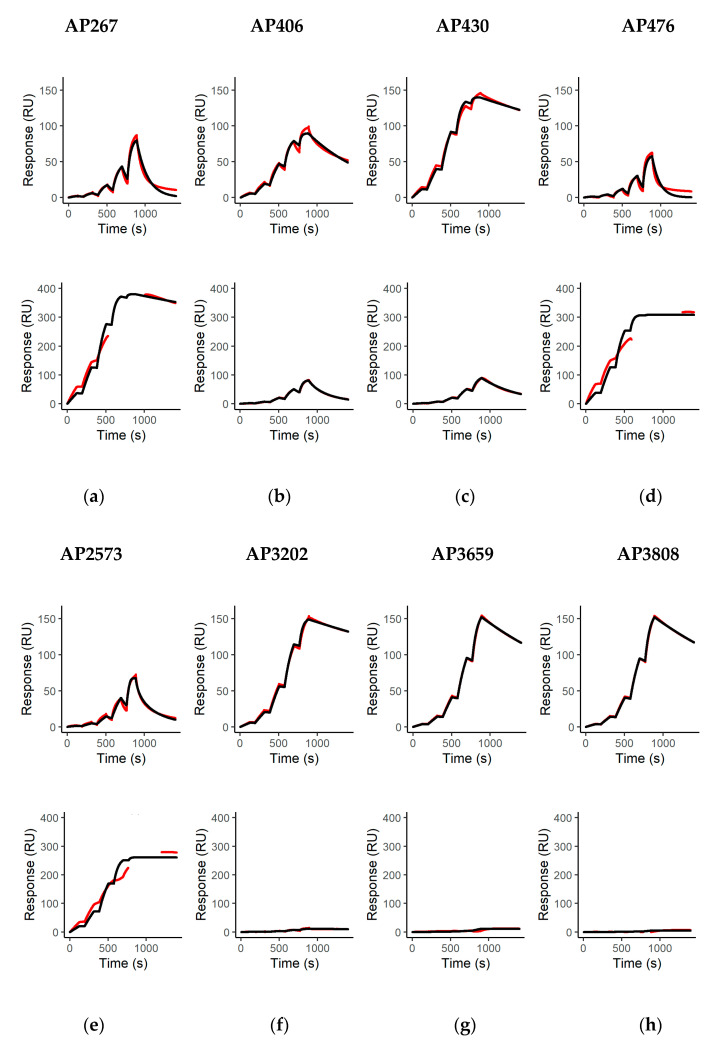
Binding kinetics of the monoclonal antibodies. Binding responses (in resonance units (RU)) of double referenced binding curves (red lines) are shown overlaid with fitting curves (black lines) from a 1:1 binding model for single cycle kinetic measurements of the indicated monoclonal antibodies (mAbs); (**a**–**h**) mAb binding either to abrin (upper panel) or binding to APA (lower panel). Five increasing concentrations of abrin or APA were injected consecutively for 120 s before a buffer was injected for 600 s after injection with the highest concentration (333.33 nM corresponding to 20 µg/mL abrin or 40 µg/mL APA, respectively).

The binding of all mAbs to their cognate antigen was further characterized by surface plasmon resonance (SPR) spectroscopy using a dilution series of abrin or APA in equimolar concentrations. SPR allowed us to determine the affinity *K_D_* as well as the binding kinetics and helped to assess the antibodies’ ability to capture native antigen from solution—a crucial prerequisite to work as capture antibodies in a sandwich ELISA or for immunoaffinity enrichment. Eight of the 15 mAbs exhibited binding to either abrin or APA or both in the SPR setting applied (Figure 2), whereas seven showed neither binding to abrin nor APA (Supplementary Figure S4). This corresponded to eight mAbs being able to capture their cognate antigen from solution, and the SPR results confirmed the preferential recognition of abrin or APA, respectively, as demonstrated previously by ELISA (Figure 1).

The eight monoclonal antibodies showing binding to either abrin and/or APA in the SPR analysis demonstrated high affinities to their cognate antigens (*K_D_* between 10^−8^ and <10^−10^ M, Table 2) with the highest affinities for abrin measured for the antibodies AP430 and AP3202 (*K_D_* 1.5 × 10^−9^ M and 3.3 × 10^−9^ M). Likewise, the antibodies AP476 and AP2573 demonstrated the highest affinities for APA (*K_D_* < 1 × 10^−10^ M).

Generally, most of the seven mAbs which did not show binding in the SPR analysis to abrin and/or APA performed well in Western blotting (AP54, AP69, AP87, AP188, AP464, and AP708; Figures S3 and S4); of these, three mAbs recognized abrin and APA equally well (AP87, AP464, and AP708), indicating a preference for the denatured antigens and/or linear epitopes. Five mAbs provided suboptimal results in Western blotting (AP12, AP406, AP2573, AP3659, and AP3808) indicating the recognition of an epitope sensitive to denaturing conditions.

The affinity *K_D_* and further characteristics of the 15 monoclonal antibodies analyzed by ELISA, Western blot and SPR are summarized in Table 2.

For selected abrin-specific mAbs, the binding specificity could be further delineated to the abrin A or B-chain by Western blotting under reducing conditions using a highly purified preparation of isolated abrin-a (devoid of abrin-b, c, and d [49,50]). As shown in Supplementary Figure S5, AP188 and AP3202 recognized an epitope on the A-chain of abrin-a, whereas AP69, AP430, AP464, and AP708 were specific for the B-chain (Table 2).

### 2.2. Establishment of Two Sandwich ELISAs for the Detection of Abrin and A. precatorius Agglutinin

**Figure 3:**
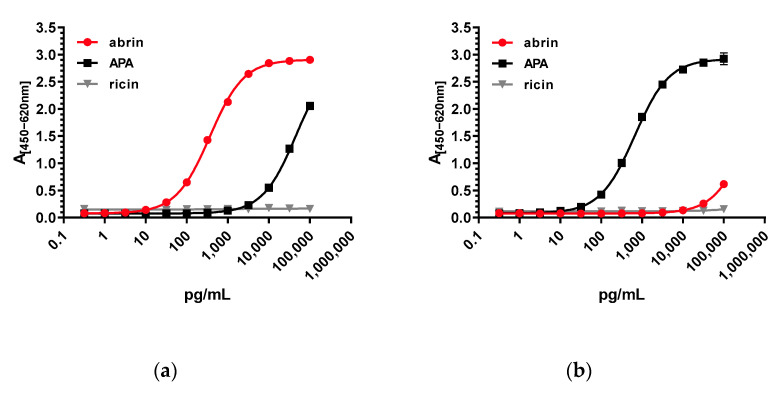
Sandwich ELISAs for the detection of abrin and APA. Serial dilutions of purified abrin (red), APA (black) as well as ricin (grey) were tested in (**a**) sandwich ELISA preferentially detecting abrin based on mAb AP430 as a capture antibody and biotinylated mAb AP3202, and (**b**) sandwich ELISA preferentially detecting APA based on mAb AP476 as a capture reagent and biotinylated mAb AP2573 as a detection reagent. Absorption was measured at 450 nm with a reference wavelength at 620 nm. Absorption was plotted against the log concentrations of the different toxins. Error bars indicate the standard deviation of two technical duplicates.

Based on the characterization of the 15 mAbs, the next step was to develop two different sandwich ELISAs to preferentially detect either abrin or APA with no cross-reactivity to the related lectins ricin and RCA120. To this end, different combinations of either abrin or APA-specific antibodies were tested. As expected, for both antigens, the best results were obtained when the mAbs with the highest affinity were combined (Table 2). For an abrin-specific ELISA with little cross-reactivity to APA, mAb AP430 was used as a capture antibody and combined with biotinylated AP3202 as a detection antibody. In order to set up an APA-specific ELISA with little cross-reactivity to abrin, AP476 was selected as a capture mAb and combined with biotinylated AP2573 as a detection antibody. The performance of the two different sandwich ELISAs against a dilution series of both abrin and APA (and the related ricin) is depicted in Figure 3. The two abrin and APA-specific sandwich ELISAs showed similar sensitivities with a half maximal effective concentration (EC_50_) of ~372 pg/mL for the abrin-specific ELISA and ~655 pg/mL for the APA-specific ELISA. Considering the twofold higher molecular weight of APA compared to abrin, this resulted in very similar molar EC_50_ concentrations.

As shown in Figure 3, the abrin-specific sandwich ELISA showed a slight cross-reactivity of approximately 0.7% towards APA; the cross-reactivity of the APA-specific sandwich ELISA with abrin was even lower (below 0.1%). No cross-reactivity towards ricin or RCA120 was observed for both ELISAs (Figure 3). Considering that abrin and APA were both purified to ≥97% from *A. precatorius* seeds, a low degree of cross-reactivity of the ELISAs was expected, and this was previously observed in ELISA for the related toxins ricin and RCA120 [48]. Indeed, a dedicated analysis by liquid chromatography coupled with electrospray ionization and tandem mass spectrometry (LC-ESI-MS/MS) of the purified toxins used as ELISA antigens identified trace amounts of APA in the purified abrin preparation and, conversely, abrin in the purified APA preparation (≤3%, see Section 2.4). Based on this, it is currently unclear if the data show a real cross-reactivity of the ELISAs or if the low amount of the respective non-target analyte is detected (e.g., for the abrin ELISA detection of the traces of abrin in the APA preparation).

#### Validation of the Abrin-Specific Sandwich ELISA

In case of a criminal or forensic investigation or to support medical diagnosis, the accreditation of the method used according to international standards ISO 15189 and ISO/IEC 17025 is desirable if not mandatory. To fulfill ISO standard criteria, the abrin-specific sandwich ELISA was comprehensively validated as described in the Materials and Methods section and finally accredited according to ISO 15189 (clinical matrices) and ISO/IEC 17025 (food, feed and environmental matrices) by the German accreditation body DAkkS.

The validation study started with the determination of the half maximal effective concentration (EC_50_) of the abrin-specific ELISA as the point of highest precision with respect to quantification and resulted in an EC_50_ of 372 ± 60 pg/mL (Table 3). As described in the Materials and Methods section, the limit of detection (LOD) was determined to be 22 ± 6 pg/mL. The working range for the quantification of the abrin-specific ELISA, as the range in which the obtained results had a coefficient of variation of ≤20% and a trueness of 80%–120%, was experimentally determined, and the lower and upper limits of quantification were determined to be 109 ± 20 pg/mL and 1270 ± 210 pg/mL, respectively. Intra-assay and inter-assay coefficients of variation of the EC_50_ concentration were determined at 8% and 12%, respectively, with *n* = 10 as the number of intra or inter-assay replicates analyzed in technical duplicates (Table 3).

While the APA-specific ELISA did not undergo a full validation, two key features were determined. The LOD was calculated from the standard curve as the mean blank reading plus 10 times the standard deviation of the blank as about 35 pg/mL and the EC_50_ value was determined at ~655 pg/mL.

**Figure 4:**
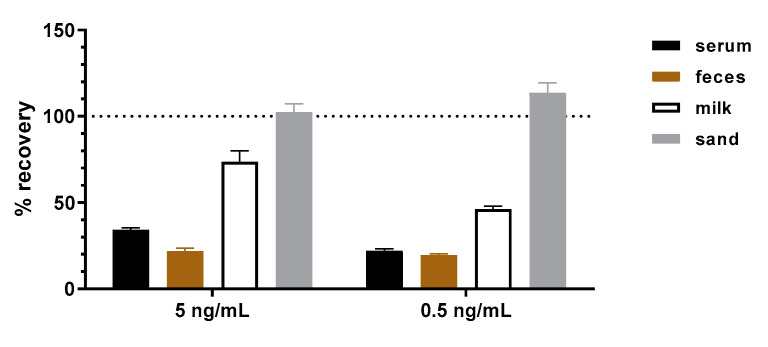
Recovery rates determined by the abrin-specific ELISA after artificially spiking abrin into four representative matrices (human serum, cat feces, semi-skimmed milk and sand). For the spiking of the toxin into the matrices, two different spiking concentrations (0.5 and 5 ng/mL) were tested. Five independent replicates were analyzed for each matrix and concentration. The concentration of abrin in each sample was determined and used to calculate the recovery rate as a percentage of the corresponding concentration spiked into the buffer, which was set to 100% (indicated by dotted line). Error bars indicate the standard deviation of the five replicates.

To assess potential matrix effects which could be encountered when analyzing clinical, food or environmental samples, four representative matrices were selected. As clinical matrices, pooled human donor serum and cat feces were analyzed. As the food matrix, semi-skimmed milk was evaluated, and as an environmental matrix, a commercially available standardized soil (100% sand) was evaluated. In the first step, the effect of the blank matrices in different amounts or concentrations without the addition of toxin on the abrin-specific sandwich ELISA was analyzed. No interfering matrix effects such as increased background signals were observed. In the second step, the influence of the matrices on the detection and quantitation of abrin was investigated. For this purpose, either the buffer or the four matrices were spiked with 5 and 0.5 ng/mL of abrin. Five independent replicates were analyzed for each matrix and concentration. The concentration of abrin in each sample was determined and used to calculate the recovery rate as a percentage of the corresponding concentration spiked into the buffer, which was set to 100%. Figure 4 shows the results for the four matrices (human serum, cat feces, semi-skimmed milk and sand) spiked with abrin. The highest recovery rate was determined with the inert matrix sand (around 100%). For milk, a recovery rate between 50% and 75% was found. Serum and feces were more challenging matrices with recovery rates between 20–30%. Different components of these two matrices—e.g., glyco-structures—seemed to mask the toxin or interfere with the toxin−antibody interaction. The matrix feces contained insoluble organic particles, so a loss of abrin binding to the particles might be an additional reason for reduced recovery.

### 2.3. Establishment of an Abrin-Specific Lateral Flow Assay (LFA) for On-Site Detection

**Figure 5:**
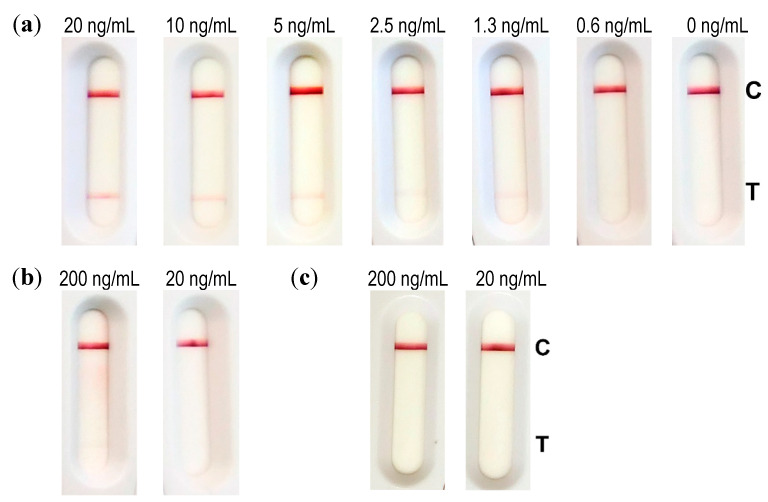
Performance of the abrin LFA for the detection of abrin spiked into buffer. The LFA was based on AP3808 as a capture antibody and colloidal gold labeled AP430 as a tracer antibody. For (**a**) abrin, 20, 10, 5, 2.5, 1.3, 0.6, and 0 ng/mL as final concentrations were used, whereas for (**b**) APA and (**c**) ricin, 200 and 20 ng/mL were tested. Abrin, APA and ricin were first diluted in 0.1% BSA/PBS followed by a 1:10 dilution in extraction buffer in accordance with the manufacturer’s instructions, and 100 µL was applied to the LFA. Results were read out after 30 min by the naked eye. C denotes the control line (anti-mouse capture antibody) and T denotes the test line (anti-abrin antibody).

For point-of-care or on-site detection, more rapid methods than stationary, lab-based ELISA are needed. To address this issue, several mAbs from Table 2. were tested as capture and as tracer antibodies on spotted strips. In total, 81 combinations were assessed for the high signal intensities achieved and potential non-specific interactions. Eight capture/tracer pairs (AP3808/AP406, AP3808/AP430, AP3808/AP3202, AP3808/AP476, AP3659/AP406, AP3659/AP430, AP3659/AP3202, and AP3659/AP476) met the defined criteria, showing a characteristic U-shape specific signal [51] and revealing a high affinity capture antibody. The three pairs of antibodies delivering the highest specific/nonspecific signal ratio were further assessed with several concentrations of abrin, and finally the best pair was determined to be the combination of AP3808 as a capture antibody and AP430 as a colloidal gold labeled tracer antibody. Notably, AP3808 was one of the three mAbs with exclusive specificity for abrin that did not bind to APA (Figure 2, Table 2). The combination AP3808/AP430 was selected for the production of industrially made LFA strips (NG Biotech, Guipry, France) which were then evaluated in an assay validation study. In the first step, the detection limit (LOD) for abrin detection and the potential cross-reactivity to APA and ricin were assessed. To this end, dilution series of abrin (20 to 0.6 ng/mL) and two concentrations (20 and 200 ng/mL) of APA or ricin were applied on different LFAs (Figure 5). The lowest concentration of abrin detected was 1.3 ng/mL, while 0.6 ng/mL of abrin could no longer be detected reliably. Therefore, the LOD of abrin spiked in buffer was considered to be ~1 ng/mL. No cross-reactivity to APA or ricin was observed even for the highest concentration tested (200 ng/mL; Figure 5).

In the second step, the performance of the LFA was analyzed for three representative matrices: namely, milk, cat feces (10% (*w*/*v*) suspension in 0.1% BSA/PBS) and sand (10% (*w*/*v*) suspension of sand in extraction buffer).

**Figure 6:**
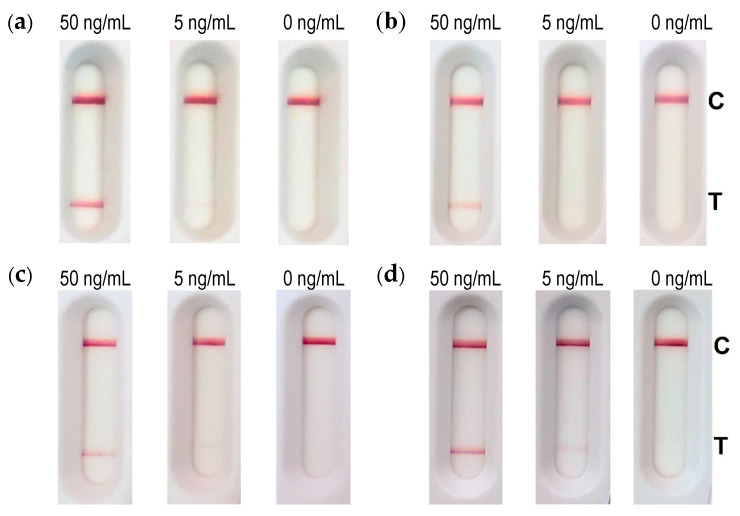
Detection of abrin in matrices using the abrin LFA. Two concentrations of abrin were spiked into (**a**) 0.1% BSA/PBS, (**b**) semi-skimmed milk and (**c**) a 10% (*w*/*v*) suspension of cat feces in 0.1% BSA/PBS, which were all further diluted with a 10-fold volume of extraction buffer resulting in the indicated final concentrations of abrin. (**d**) A 10% (*w*/*v*) suspension of sand in extraction buffer was spiked with abrin at 50 and 5 ng/mL and the sand was removed by centrifugation. Then, 100 µL of each solution was applied to the sample well. Results were read out after 30 min by the naked eye. C denotes the control line (anti-mouse capture antibody) and T denotes the test line (anti-abrin antibody).

Milk, the cat feces suspension (in 0.1% BSA/PBS) and 0.1% BSA/PBS as buffer control were spiked with abrin, and after 10-fold dilution in extraction buffer corresponding to a final concentration of 50 or 5 ng/mL abrin, 100 µL was applied to the sample well of the LFA. Sand was mixed with a 10-fold volume of extraction buffer and spiked with 50 and 5 ng/mL abrin before loading 100 µL on the LFA. Figure 6 shows that 50 ng/mL abrin was readily detectable from all matrices tested, while the lower concentration of 5 ng/mL was still visible from buffer, cat feces and sand but hardly detectable from milk, indicating a loss in sensitivity here.

### 2.4. Application of the Monoclonal Antibodies for LC-ESI-MS/MS with Immuno-Enrichment

Generally, immunological methods based on highly specific and affine antibodies offer several advantages such as exquisite sensitivity and compatibility with routine applications, but they usually do not deliver unambiguous results. Here, MS-based techniques are clearly advantageous as they enable the unambiguous identification of a protein based on its peptide fingerprint—an issue that is highly relevant in the course of a forensic investigation [52]. Additionally, MS-based methods can deliver detailed information on known and even unknown sample contents, especially when using scanning-mode MS approaches, thereby adding an open view to the diagnostic workflow [53]. Moreover, absolute quantification can be achieved by spiking stable-isotope-labeled peptides to the samples. However, MS methods can be severely hampered by the presence of other peptides or proteins in excessive amounts. Thus, affinity purification steps prior to MS analysis have been used to extract the target analyte(s) from complex sample matrices. In this context, we have recently published two manuscripts applying the antibodies described in this work for immuno-affinity enrichment followed by the LC-ESI-MS/MS-based or MALDI-TOF-based identification and quantification of abrin in complex matrices [42,43]. Based on our previous work, we identified a combination of four mAbs—namely AP430, AP3808, AP3659—directed against abrin plus AP476 specific for APA, as an optimal mixture for the immuno-enrichment of both abrin and APA from samples, followed by a tryptic digest and MS analysis.

In the context of the current work, we conducted a trace analysis study, using the purified abrin and APA preparations reported herein to characterize the antibodies’ binding profiles (Figure 1, Figure 2 and Figure 3). The question was if low amounts of APA in excessive amounts of abrin and, conversely, abrin in APA could be clearly identified considering their high identity on the amino acid level (74.8%; Table 1). To this end, approximately 80 µg of the abrin or the APA preparation was subjected to immuno-enrichment using the previously established protocol followed by tryptic digestion and LC-ESI-MS/MS analysis. As shown in Supplementary Figure S6, all four isoforms of abrin (abrin-a to d [26]) were identified and sequenced with a sequence coverage of 53% to 60% in the purified abrin preparation. Despite the high purity of the abrin preparation (>97%), peptides specific for APA could be identified and sequenced with a sequence coverage of 39% indicating a low cross-contamination of abrin with APA.

Likewise, the same procedure applied to the purified APA preparation delivered 52% sequence coverage for APA-specific peptides (Supplementary Figure S7). Additionally, a low amount of cross-contaminating abrin (isoforms abrin-a, b, and d) with a sequence coverage of 43% to 54% was identified as well, accounting for some 2%–3% of the material (Supplement Figure S7).

### 2.5. Application of the Monoclonal Antibodies in Diagnostics of an Attempted Suicide Case

**Figure 7:**
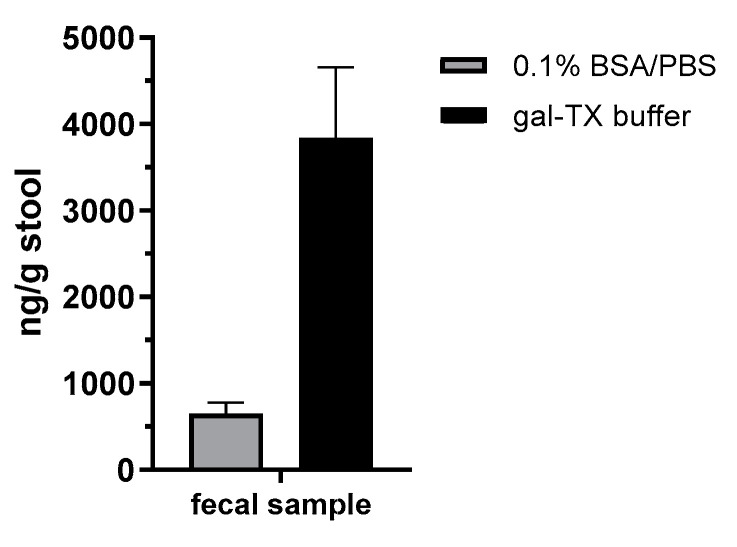
Analysis of a fecal sample from a suicide attempt with *Abrus precatorius* using abrin-specific ELISA. Stool was extracted for 30 min 1:5 (*w*/*v*) with either 0.1% BSA/PBS or with the same buffer supplemented with 1% Triton X-100 and 250 mM galactose. Suspension was clarified by centrifugation and supernatants were measured applying the abrin-specific ELISA. Quantitation was based on a standard curve using the purified abrin preparation described in this work.

In order to demonstrate the diagnostic value of the different methodologies established in this work, we applied both the abrin-specific ELISA and the LC-ESI-MS/MS approach in an attempted suicide case with oral *A. precatorius* uptake in Germany. Here, a single human fecal sample was obtained approximately 24 h after the ingestion of at least one chewed *A. precatorius* seed (according to the patient). The stool was extracted for 30 min with either 0.1% BSA/PBS or 0.1% BSA/PBS containing 1% (*v*/*v*) Triton X-100 and 250 mM galactose (gal-TX buffer) to increase toxin recovery by facilitating desorption from glyco-structures. The extracts were clarified by centrifugation and analyzed using the abrin-specific ELISA (Figure 3a). As shown in Figure 7, ELISA quantification provided about 660 ng abrin per gram of stool in the 0.1% BSA/PBS buffer extract, but 3840 ng abrin per gram of stool in the gal-TX buffer, indicating a 5.8-fold increase in toxin recovery from the challenging matrix feces when using a galactose/detergent-containing extraction buffer.

**Figure 8:**
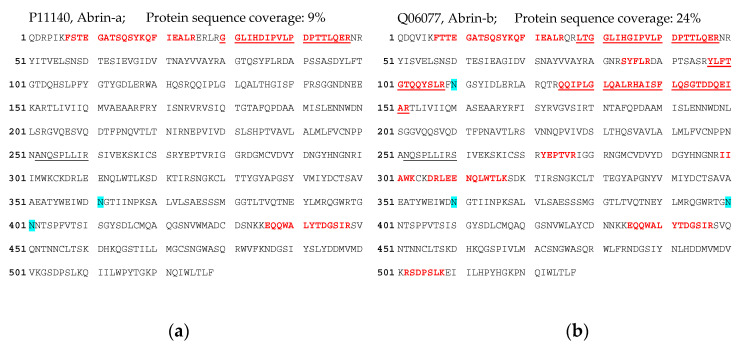
Protein sequence coverage of proteins identified in a human fecal sample from a suicide attempt after immuno-affinity enrichment, tryptic digest and non-targeting liquid chromatography coupled with electrospray ionization and tandem mass spectrometry (LC-ESI-MS/MS) analysis. Sequences of identified abrin isoforms (**a**) abrin-a (UniProt P11140) and (**b**) abrin-b (Q06077) are shown after a MASCOT server search against a self-assembled UniProt/NCBI database containing all abrin isoforms and *Abrus precatorius* agglutinin as well as an NCBI database containing all *Abrus precatorius* proteins. Amino acids highlighted in red were experimentally identified with a sequence coverage of 9% for abrin-a and 24% for abrin-b. Underlined peptides represent proteotypic peptides for (**a**) abrin-a or (**b**) abrin-b, respectively. Asparagine (N), highlighted in turquoise, represents potential N-linked glycosylation sites. The linker peptide sequence between the two chains of both abrin isoforms is underlined in black.

In order to confirm the presence of abrin in the fecal sample, an immuno-affinity enrichment protocol was applied to the stool extract, followed by a tryptic digest and LC-ESI-MS/MS detection (Figure 8). In this challenging clinical matrix, abrin could be identified with a sequence coverage of 24% for the isoform abrin-b and 9% for abrin-a, confirming the previous ELISA results.

## 3. Discussion

In the current work, a panel of 15 mAbs specific for either abrin, APA or both was generated and comprehensively characterized by ELISA, SPR and Western blotting, and suitable mAbs were implemented into sandwich ELISA, LFA and MS-based approaches. Key features of the methodologies were highlighted and the approaches were applied to analyze representative complex clinical, food and environmental matrices, including a clinical sample from a human case of *A. precatorius* intoxication. An overview of the use of the different mAbs in the different applications is given in Table 4, with antibodies depicted in bold showing the superior performance when applied in the indicated methods.

Antibodies are still the most versatile tools to specifically detect their target molecule in a broad range of matrices. Here, mAbs in comparison to polyclonal antibodies (pAbs) have been shown to offer the advantage of defined specificity and often higher sensitivity, provided that high-affinity mAbs are used. In terms of quality management, mAbs derived from stable hybridoma clones can be produced with a constant quality over time, thus increasing the reproducibility of experimental data and preventing the lot-to-lot variability observed with pAbs. In line with recommendations on mAb validation [54,55], the mAbs described in this work were characterized to demonstrate their fitness for purpose, assessing their target antigen, binding selectivity (cross-reactivity), binding strength (affinity) and the influence of non-target substances (matrix effects). Not surprisingly, different mAbs turned out to be optimal for different applications, with some of them targeting epitopes on the native antigen, making them suitable tools for sandwich ELISA or immuno-affinity enrichment, and others targeting denatured epitopes relevant in Western blotting (Table 4). Interestingly, the panel of mAbs comprised antibodies showing a strong preference for either abrin (AP3202, AP3659, AP3808), for APA (AP2573) or both (AP87, AP464, AP708), with the latter three applicable only in Western blotting and indirect ELISA. By carefully selecting highly affine mAbs that preferentially detect abrin over APA or vice versa, two ELISA systems were developed in this work which allowed us to discriminate between purified abrin and purified APA, with less than 0.7% (abrin-specific ELISA) or 0.1% (APA-specific ELISA) cross-reactivity between the two related lectins. It is worthy of note that by combining a capture mAb directed against the B-chain of abrin (AP430) with a detection mAb recognizing the A-chain (AP3202), the abrin-specific ELISA detected only the intact A-B toxin. In previous works, the issue of mAb / ELISA selectivity for abrin versus APA has rarely been addressed; only a few groups have reported either cross-reactive or specific mAbs for abrin and APA derived from mouse or llama [32,56,57]. In a broader context, sandwich ELISAs able to differentiate between related toxin subtypes or isolectins have been successfully established based on highly specific mAbs directed against unique domains found in the related molecules (e.g., for ricin/RCA120 or for the related botulinum neurotoxins BoNT/C, CD, DC, and D [47,48]). Additionally, based on specific mAbs, a surface plasmon resonance sensor has been developed to simultaneously differentiate and quantify ricin from RCA120 in real time in less than 30 min [58]—an application that is now open to be explored for abrin and APA as well.

In comparison, the pAb described here, KAP142, was unable to distinguish between the select agent abrin and the related APA, which are 74.8% identical at amino acid level, thus preventing its use in criminal or forensic investigations where discrimination between these two molecules is mandatory. Even worse, KAP142 also reacted with the related plant lectins ricin and RCA120, which share 48.8% or 44.6% sequence identity with abrin, respectively. This type of cross-reactivity between abrin/APA and ricin/RCA120 has previously been observed for other pAbs [30,59,60]; it has even been reported that mAbs —similarly to pAbs—showed cross-reactivity between abrin and ricin [61,62]. Interestingly, these mAbs were derived from naïve human or llama phage-display libraries and not from an immunized host.

With respect to matrix interference, pAbs are more likely to react with non-target substances in complex samples, which often results in elevated background signals [31]. This is due to their polyclonal nature and the presence of antigen-unrelated antibodies. The application of the mAbs presented in this work in a sandwich ELISA to detect abrin from representative clinical (serum, feces), food (milk) and environmental (sand) samples did not result in an elevated background. When abrin was spiked into the four representative matrices, ELISA results delivered recovery rates between 20–110%. It is well known that matrix effects play a major role in assay performance [46,63,64,65] since components of the matrix can interfere with the non-covalent interactions between antibodies and antigens stabilized by electrostatic forces, hydrogen bonds, van der Waals forces and/or hydrophobic forces. Additionally, matrix components might mask or expose antibody-binding epitopes, leading to decreased or increased antibody–antigen binding. The difficulty is that the extent to which these effects occur with different matrices cannot be anticipated but has to be assessed empirically. Therefore, although challenging matrices have been tested, the validation study initiated in this work has to be extended in the future to include a broader spectrum of clinical, food and environmental matrices.

Regarding assay sensitivity, the two stationary ELISAs developed in this work delivered excellent detection limits: for the abrin-specific ELISA, an LOD of 22 pg/mL, and for the APA-specific ELISA, an LOD of ~35 pg/mL were determined. In comparison, previously reported ELISAs for abrin delivered detection limits between 100 pg/mL and 7800 pg/mL [31,32,33,34,57]; therefore, the current work significantly advances the field, offering tools with higher sensitivity as well as increased specificity and selectivity.

In a potential biothreat scenario, the rapid detection of threat agents is beneficial to support an immediate risk assessment and to protect first responders entering the scene. In this situation, a stationary ELISA as described above, although highly sensitive and specific, turns out to be of limited use, due to its long assay time (4–6 h) and the requirement of a laboratory surrounding with trained personnel. To address this, LFAs and biosensors have been developed and optimized for use by non-trained personnel in the field, delivering results in less than one hour [34,35,36,37]. The mAbs described in this work were tested and incorporated into LFA cartridges at CEA and were selected for industrial production with a commercial partner. Unlike most other LFAs, the LFAs established here preferentially detected abrin over APA (LOD ~1 ng/mL) and as such would provide first responders with a more robust risk assessment compared to cross-reactive LFAs. To date, there is only one other product described which is able to discriminate between abrin and APA [34]. Due to the lack of washing steps, LFAs might encounter problems with complex matrices. To address this, the LFA was tested with the representative matrices of milk, feces and sand. Concentrations of 5–50 ng/mL could still be detected, which was well in the range of 0.3 to 50 ng/mL reported for other LFAs [34,36,37]. It should be noted that, in the case of fecal samples, feeding, drinking and other living conditions will presumably alter the extraction efficacy to lower or higher rates, meaning that the sensitivity cannot generally be anticipated.

Apart from methods targeting the toxin itself, techniques addressing the biological activity of abrin are important to assess the threat potential in an incident and are required to complement a comprehensive analysis of evidences in a criminal or forensic investigation. As described above, our sandwich ELISA detects both the A and the B chain present in intact abrin. However, this is not yet sufficient evidence of biological activity. In order to assess the toxin’s functional activity, in vivo experiments, cell-based cytotoxicity assays and assays measuring the depurination of the rRNA can be performed—approaches that have been previously described for ricin as well [66,67,68]. As both toxins result in similar biological responses based on the same functional mechanism within the cell, a discrimination between abrin and ricin by the use of specific neutralizing antibodies is important [68,69].

In this work, a major factor to delineate the specificity and selectivity of the mAbs and the corresponding assays was access to highly purified abrin (containing all four isoforms abrin-a, b, c, and d) and APA preparations. Actually, the separation of the four abrin isoforms from APA is challenging, since they share a high sequence identity (see above) and run at similar molecular weights in an SDS-PAGE assay under non-reducing conditions [50]. Based on previous publications [50], an optimized purification protocol was developed that delivered abrin and APA preparations of an estimated purity ≥97% as determined by MALDI-TOF MS and SDS-PAGE [45]. This protocol will serve as a starting point to further develop a candidate reference material for abrin in the current European project EuroBioTox [70], which aims at establishing validated procedures for the detection and identification of biological toxins, including the plant toxins abrin and ricin. The challenges ahead in producing and characterizing certified reference materials have recently been summarized by the consortium [71]. In the context of reference material production, one critical issue is that the main component(s) as well as any impurities have to be identified and quantified by a combination of biochemical, immunological and spectrometric methods [48,71]. In preparation for this endeavor, we performed a trace analysis applying an immuno-affinity enrichment protocol followed by a tryptic digest and LC-MS/MS analysis developed on the basis of our mAbs [42,43]. Starting with the purified abrin and purified APA preparations, it was our goal to determine if low amounts of APA in excessive amounts of abrin and, conversely, of abrin in APA could be clearly identified considering their high identity on the amino acid level. Indeed, the analysis showed that both the main component(s), and even the impurities which accounted for below ≤3% could be identified by LC-ESI-MS/MS with a high sequence coverage (52%–60% sequence coverage for the main component(s) and 39–54% sequence coverage for the impurity). This will serve as starting point for a more comprehensive characterization of the future abrin reference material; e.g., by applying MS-based quantification of the components based on labeled AQUA peptides [42,43].

In order to demonstrate the diagnostic value of the sandwich ELISA and the immuno-affinity enrichment LC-ESI-MS/MS approach based on the mAbs described in this work, we applied the methods in an attempted suicide case with oral *A. precatorius* uptake in Germany. Here, human feces were obtained approximately 24 h after ingestion of at least one chewed *A. precatorius* seed according to the patient’s statement. In order to optimize sample preparation, we tested two buffers: one containing 0.1% BSA/PBS, the other one 0.1% BSA/PBS plus 1% (*v*/*v*) Triton X-100 and 0.25 M galactose. For the latter buffer, we took advantage of previous works in animal models of ricin intoxication, where either 0.25 M of lactose or galactose was used for sample preparation [72,73]. Ricin and abrin as lectins both bind to tissue or matrix components containing carbohydrates, so a high concentration of galactose in the homogenization buffer was thought to aid detachment [72]. Indeed, we obtained a 5.8-fold increase in abrin recovery from the challenging matrix feces when the galactose/detergent-containing extraction buffer was used for ELISA quantification. Notably, the ELISA results could be confirmed by the immuno-affinity enrichment LC-ESI-MS/MS approach, delivering five proteotypic peptides for abrin-a (one peptide) and b (four peptides). The sequence coverage obtained was, as expected, low but still enabled unambiguous identification with 9% sequence coverage for abrin-a and 24% for abrin-b; the isolectins abrin-c and d could not be detected. In order to put these results into perspective with other cases of *A. precatorius* intoxication worldwide, we performed a literature search for case descriptions of *A. precatorius* intoxication. Since 1961, we found 23 case descriptions of human *A. precatorius* intoxications in the literature, either linked to accidental, voluntary or suicidal uptake (22 cases of oral uptake, one case of injectional uptake). For the majority of cases, the link to *A. precatorius* was demonstrated by circumstantial evidence based on details of the case report; e.g., known or observed uptake of plant seeds or the finding of plant material, but explicitly not by the detection of abrin. In four out of 23 cases, diagnostic assays successfully detected and identified the low molecular weight molecule *L*-abrine (*N*-methyl-L-tryptophan) in urine as a surrogate marker for abrin intoxication [18,19,20,74]. In none of the cases was abrin itself detected. Therefore, to the best of our knowledge, this is the first case where ELISA-based detection and quantification as well as LC-ESI-MS/MS-based identification were successfully implemented for abrin detection in a real-life case of human *A. precatorius* intoxication.

## 4. Materials and Methods

### 4.1. Toxins

All toxins as well as the mixture of *A. precatorius* lectins were handled by trained personnel under a class II vertical laminar flow cabinet (Heraeus Herasafe, Thermo Scientific, Dreieich, Germany) in a dedicated toxin laboratory. Toxin-containing solutions were inactivated with sodium hydroxide in a final concentration of 5% overnight, and solid waste containing traces of toxin was inactivated by autoclaving (134 °C, 1 h).

Purified abrin, purified APA, a mixture of *A. precatorius* lectins, ricin and *R. communis* agglutinin were all produced in-house. Ricin and RCA120 were purified from the seeds of *R. communis* variety *carmencita pink* similar to protocols described earlier [30,69,75]. The material was extensively characterized and purity was determined as ≥97% [48]. Abrin and APA were purified and separated from each other following a similar strategy. The purity of both preparations was determined by mass spectrometry as ≥97% (Supplementary Figures S1 and S2) [48,73].

For the purified mixture of *A. precatorius* lectins, proteins with lectin properties were purified from the extract of *A. precatorius* seeds by affinity-chromatography using an XK16/20 column (Cytiva, Freiburg, Germany) packed in-house with lactosyl-sepharose. Analysis by MALDI-TOF MS showed the presence of abrin and APA in an estimated ratio of 2:3.

### 4.2. Matrices

A human serum pool was kindly provided by MH Hannover, Germany, and semi-skimmed milk was bought from a local retail store. Artificial soil #2 (100% sand, standardized reference soil) was obtained from Ros Consulting and Development AB, Sweden. For reconstitution 20 g of dry sand was mixed with 2 mL of distilled water by rotation overnight at 4 °C until sand was completely wetted. Cat feces was collected, autoclaved at 120 °C, homogenized in 0.1% BSA/PBS pH 7.4 (1:10) and filtered through a 212 µm sieve to remove residual fur. The 10% cat feces suspension was used for the spiking experiments of matrices.

### 4.3. Clinical Sample Material of an A. precatorius Intoxication Case

A fecal sample of an *Abrus precatorius* intoxication was homogenized to a ratio of 1:5 (*w*/*v*) with assay buffer (PBS, 0.1% BSA) or gal-TX buffer (PBS, 0.1% (*w*/*v*) BSA, 0.25 M galactose and 1% (*v*/*v*) Triton X-100) and extracted under 30 min of shaking at 4 °C followed by a centrifugation step (5 min, 12,000× *g*, 4 °C). The supernatant was used for analysis in the abrin-specific ELISA and immuno-enrichment for LC-ESI-MS/MS analysis. For the abrin-specific ELISA, the supernatants were used undiluted and diluted further with assay buffer.

For immuno-enrichment, 250 µL of sample (extract containing assay buffer) was taken for immuno-affinity-enrichment using mAb AP3202 and AP3808 coupled to magnetic Dynabeads^®^ (Invitrogen, Karlsruhe Germany) and on-bead tryptic digest (0.5 µg trypsin, 120 min at 37 °C) followed by non-targeting LC-ESI-MS/MS analysis (for details, see the section on Mass Spectrometry).

### 4.4. Sequence Analysis

For sequence comparison, the following protein sequences from the UniProt database were used: abrin-a, ABRA_ABRPR/P11140; *Abrus precatorius* agglutinin, AGGL_ABRPR/Q9M6E9; ricin, RICI_RICCO/P02879; and *Ricinus communis* agglutinin, AGGL_RICCO/P06750. Sequences were uploaded into the Geneious Prime 2020.2 software package (Biomatters Ltd., Auckland, New Zealand). Protein sequences were aligned and distances calculated using MUSCLE Alignment (3.8.425) with a maximum of eight iterations.

### 4.5. Generation of Monoclonal and Polyclonal Antibodies

Handling of laboratory animals was performed in compliance with the regulations of the German Animal Welfare Act and European legislation for the protection of animals used for scientific purposes (Directive 2010/63/EU). Immunizations of mice to generate mAbs were approved by the State Office for Health and Social Affairs in Berlin (LAGeSo Berlin, Germany) under the registration numbers H129/19 and H109/03. Sacrifice of mice for the removal of thymocytes was registered by the LAGeSo under the number T0060/08.

Monoclonal antibodies (mAb) were generated as described previously [46,47,76]. In brief, BALB/c or NMRI mice (Charles River, Sulzfeld, Germany) were used at the age of 8 weeks. Three female mice were immunized with ~50 µg of a mixture of *A. precatorius* lectins inactivated by formaldehyde. The inactivated mixture of *A. precatorius* lectins was prepared by adding 37% formaldehyde to the mixture, resulting in a final concentration of 0.5% formaldehyde, followed by incubation for 21 days at 37 °C. Mice were boosted several times with similar doses of the same antigen in adjuvant at four-week intervals. Once animals had mounted a substantial titer, they were boosted with 5 µg of the native *A. precatorius* lectin mixture in adjuvant. On day −3, −2 and −1 before fusion, 5 µg of the native *A. precatorius* lectin mixture in phosphate-buffered saline (PBS) was applied intraperitoneally. Hybridomas were produced by fusing spleen cells from immunized mice with myeloma cells (P3-X63-Ag8.653, American Type Culture Collection) at a ratio of 2:1 in polyethylene glycol 1500 (PEG, Roche Diagnostics, Mannheim, Germany). Cells fused together with thymocytes as feeder cells were grown in selective RPMI1640 media containing 20% (*v*/*v*) fetal bovine serum, 5.78 µM azaserine, 100 µM hypoxanthine, 50 µM 2-mercaptoethanol and 500 U/mL murine interleukin-6 (IL-6). Hybridoma supernatants were screened by an indirect ELISA against the *A. precatorius* lectin mixture and additionally against ricin/RCA120 to exclude cross-reactive antibodies at days 10 to 14 post-fusion. Positive hybridoma clones were subcloned at least twice. Immunoglobulins (IgG) were purified from hybridoma supernatants grown in RPMI media supplemented with IgG-free fetal bovine serum by affinity chromatography over a HiTrap MabSelect SuRe column using a Fast Protein Liquid Chromatography System (ÄKTA, GE Healthcare Bio-Sciences AB, Uppsala, Sweden). The isotype of all purified monoclonal antibodies (mAbs) was determined using an antibody isotyping sandwich ELISA (antibodies and controls from SouthernBiotech/Biozol Diagnostica Vertrieb, Eching, Germany).

Purified antibodies were coupled to biotin according to the manufacturer’s instructions (EZ-Link Sulfo-NHS-LC-biotin; Pierce, Rockford, IL, USA). Biotinylated antibodies were stored in PBS with 0.2% (*w*/*v*) bovine serum albumin (BSA; Serva, Heidelberg, Germany) and 0.05% (*w*/*v*) NaN_3_ (Carl Roth, Karlsruhe, Germany).

Generation of pAb: polyclonal antibodies were generated in white New Zealand rabbits immunized subcutaneously with ~50 µg of a mixture of *A. precatorius* lectins inactivated by formaldehyde. Blood was collected every four weeks after the second booster immunization for serum preparation. Serum was affinity purified over a protein G column on an ÄKTA LC-instrument to obtain the IgG fraction (ÄKTA, GE Healthcare Bio-Sciences AB, Uppsala, Sweden).

### 4.6. Indirect ELISA and Sandwich ELISA

For indirect ELISA, MaxiSorp microtiter plates (Nunc MaxiSorp flat bottom, Thermo Scientific, Dreieich, Germany) were coated with abrin, APA, ricin or RCA120 at 500 ng/mL in 50 μL PBS/1 µg/mL BSA overnight at 4 °C and blocked with 2% skimmed milk in PBST (phosphate buffered saline with 0.05% (*v*/*v*) Tween 20, Merck, Darmstadt, Germany) for 1 h at room temperature. After washing, 50 µL of antibody (10 µg/mL anti-abrin/APA antibodies or anti-ricin/RCA120 antibody R109), or in the initial hybridoma screening, 50 µL of undiluted hybridoma supernatant, was added and incubated for 1 h at room temperature. After washing, the ELISA was developed by incubation with horseradish peroxidase (HRP)-labeled goat anti-mouse IgG (Fc-γ specific; Dianova, Hamburg, Germany; for detection of mouse mAbs) or HRP-labeled goat anti-rabbit IgG (Dianova, Hamburg, Germany; for detection of polyclonal antibody KAP142) diluted in 2% skimmed milk in PBST (30 min, room temperature), followed by washing and incubation with substrate 3,3′,5,5′-tetramethylbenzidine (TMB, SeramunBlau slow2 50, Seramun Diagnostika, Heidesee, Germany). The color reaction was stopped with 0.25 M sulfuric acid and the absorption was determined at 450 nm (referenced to 620 nm) using a microtiter plate reader (Infinite M200, Tecan, Männedorf, Switzerland).

For sandwich ELISA, MaxiSorp microtiter plates were coated with 5 µg/mL of primary mAb AP430 (abrin-specific ELISA) or AP476 (APA-specific ELISA) in 50 µL of PBS overnight at 4 °C and blocked with casein buffer (Senova, Jena, Germany) for 1 h at room temperature. After washing, 50 µL of toxin was added in serial dilutions from 100 ng/mL to 0.3 pg/mL in assay buffer (PBS, 0.1% BSA (Sigma-Aldrich, Munich, Germany)) and incubated for 2 h at room temperature. After washing, the sandwich ELISA was developed by incubation with biotin-labeled secondary antibody (AP3202 for abrin-specific ELISA; AP2573 for APA-specific ELISA) diluted in casein buffer (1 h, room temperature), followed by washing and detection with Streptavidin-PolyHRP40 (0.5 ng/mL, Senova, Jena, Germany) and substrate TMB. The color reaction was stopped with 0.25 M sulfuric acid and the absorption was determined at 450 nm (referenced to 620 nm) using a microtiter plate reader.

### 4.7. Validation of Sandwich ELISA

For statistical analysis, the standard curve of the abrin-specific sandwich ELISA was measured in 10 independent runs over 10 days, with two technical replicates per concentration. Differences in absorbance at 450 nm and reference wavelength at 620 nm were plotted against the logarithmic concentration of the abrin standard and fitted against a sigmoidal dose–response curve (four-parametric non-linear regression analysis) in Prism 8.4 (GraphPad, La Jolla, CA, USA).

The limit of detection (LOD) was calculated from the regression curve by using the absorption value (LOD(A_450–620 nm_)) calculated according to Equation (1) [77,78]. The limit of blank (LOB) determined was the mean absorbance (A_450–620 nm_) of 178 determinations of blanks. For the calculation of the standard deviation of low concentrations, in total 80 determinations of the absorbance of low concentrated samples were performed.

LOD(A_450–620 nm_) = LOB + 1.645 × SD(LowC) (1)

where LOD is limit of detection, LOB is limit of blank, SD is standard deviation and LowC is low concentration.

The lower and upper limits of quantification (LLOQ and ULOQ) were calculated based on the sigmoidal standard curve. The LLOQ and ULOQ flank the linear range of the sigmoidal curve between the inflection points of the first derivative of the sigmoidal regression curve and were computed as the maxima and minima of the second derivative.

The intra-assay coefficient of variation (CV%_intra_) at the half maximal effective concentration (EC_50_) was determined as the standard deviation divided by the mean concentration of 10 double determinations of the EC_50_ within plates multiplied by 100.

The inter-assay coefficient of variation (CV%_inter_) at the EC_50_ was calculated in relation to the concentrations determined between 10 separate and independent runs, measured in technical duplicates.

The recovery of abrin in different matrices with the abrin-specific abrin ELISA was evaluated by analyses of selected matrices spiked with two concentrations of abrin (5 and 0.5 ng/mL) or without toxin (blank matrices). As a spiking control, buffer (0.1% BSA/PBS) was spiked with the same concentrations and treated as the spiked matrices. Buffer, human serum pool, semi-skimmed milk and 10% (*w*/*v*) cat feces suspensions were spiked directly with abrin (1:100 ratio toxin/matrix). The wettened sand was first resuspended in buffer and spiked with abrin. Afterwards, spiked and blank matrices were incubated under rotation for 30 min at 4 °C followed by centrifugation for 3 min with 12,000× *g*. Supernatants were analyzed by abrin-specific ELISA. Five independent replicates were prepared and analyzed for each matrix and concentration. The concentration of abrin in each sample was determined and used to calculate the recovery rate as a percentage of the corresponding concentration spiked into buffer, which was set to 100%.

### 4.8. SDS-PAGE

In total, 2 µg abrin and APA were separated on a 12% SDS-PAGE under non-reducing conditions, or 15 µg abrin-a was separated on a 12% SDS-PAGE under reducing conditions, respectively, followed by staining with colloidal Coomasssie Brilliant Blue overnight. Images were captured by a CCD camera (ChemiDoc, BioRad, Feldkirchen, Germany).

### 4.9. Western Blot

In total, 100 ng abrin, APA, ricin or BSA were separated on a 12% SDS-PAGE under reducing conditions and transferred onto an Immuno-Blot 0.45 μm PVDF membrane (Invitrogen, Karlsruhe, Germany). After blocking the membrane in blocking buffer (2% skimmed milk in PBST) at 4 °C overnight, diluted primary anti abrin/APA antibodies (final concentration 5 µg/mL) in blocking buffer were added to the membrane for 1 h. After three washing steps, the membrane was incubated with biotin-labeled goat anti-mouse IgG (1:5000; Dianova, Hamburg, Germany; for detection of mouse mAbs) or biotin-labeled goat anti-rabbit IgG (1:5000; Dianova, Hamburg, Germany; for detection of polyclonal antibody KAP142) in blocking buffer at room temperature for 30 min and was developed with avidin–alkaline phosphatase (incubation for 20 min, final concentration 0.5 µg/mL in PBST; Avidx™-AP, Fisher Scientific, Bremen, Germany) and the chemiluminescent substrate CDP-Star (Perkin Elmer, Waltham, MA, USA). Images were captured by a CCD camera (ChemiDoc, BioRad, Feldkirchen, Germany).

### 4.10. Surface Plasmon Resonance (SPR) Measurements

The affinity and kinetics of all mAbs for binding to abrin (molecular weight approximately 60 kDa) and APA (molecular weight approximately 120 kDa) were determined as described previously with minor modifications [58]. Briefly, a series S sensor chip CM5 was modified with rabbit anti-mouse antibodies using the mouse antibody capture kit and the amine coupling kit (all Cytiva, Freiburg, Germany) according to the manufacturer’s instructions. The rabbit anti-mouse antibody bound all IgG subclasses used in this work equally well and showed a highly stable binding of the captured antibodies. Thus, the determined binding kinetics were not affected by the antibody isotype and/or drifting baselines by instable capturing. Before usage, the modified sensor chip was conditioned by injections of purified polyclonal mouse IgG (Dianova, Hamburg, Germany) at 100 µg/mL for 300 s at a flow rate of 5 µL/min over all four flow cells (Fc) and regeneration using injections of 10 mM glycine/HCl buffer at pH 1.7 (Cytiva) at 10 µL/min for 180 s, repeated three times. All measurements were performed on a Biacore T200 (Cytiva) at 25 °C using HBS-EP+ buffer (10 mM HEPES, pH 7.4, 150 mM NaCl, 3 mM EDTA, 0.05% (*v*/*v*) Tween 20) supplemented with 10 mg/mL D-galactose (Carl Roth, Karlsruhe, Germany) to suppress the unspecific binding of abrin or APA with immobilized antibodies due to their lectin activity. MAbs were diluted to 2 µg/mL and captured on Fc 2 and 4 for 60 s at a flow rate of 5 µL/min leading to ligand immobilization levels between approximately 190 and 350 resonance units (RUs). Binding kinetics were determined using single-cycle kinetics by injecting increasing concentrations of abrin or APA in a 1:3 dilution series ranging from 4.17 nM to 333.33 nM (20 µg/mL abrin or 40 µg/mL agglutinin). Association was monitored by injecting abrin or APA for 120 s, while dissociation was monitored for 600 s by injecting buffer at flow rates of 30 µL/min. Regeneration between runs was performed as described above using 10 mM glycine/HCl buffer at pH 1.7. Before and after each measurement, buffer was injected over immobilized mAbs as blank measurements, which were used for double referencing binding curves [79]. To determine kinetic binding parameters, a 1:1 Langmuir binding model (A + B = AB) with R_max_ fitted globally and an RI set to 0 was fit to the measured binding curves using the Biacore T200 Evaluation Software Version 3.2 (Cytiva, Freiburg, Germany). For APA, instead of blank measurement with buffer injections, binding curves after injection of APA over non-binding mAbs (AP12, AP54, AP69, AP87) were used as blank measurements. This was done due to the high lectin-mediated binding of APA to both control (FC 1 and 3) and measurement flow cells (FC 2 and 4), leading to artefacts in the binding curves after subtracting the signals from Fc 1 from 2 and Fc 3 from 4. Although this efficiently solved the issue of artificially distorted binding curves in the lower concentration range, distortion was still visible in the higher range (111.11 and 333.33 nM), which is why those concentrations were excluded from the analysis for curve fitting. Mean binding kinetics and affinities were determined and calculated from two technical replicate measurements for all mAbs tested.

### 4.11. Lateral Flow Assay

#### 4.11.1. Evaluation of Antibodies for Lateral Flow Assay

To select the best mAb pairs, and in order to develop a two-site lateral flow test, a combinatorial analysis was carried out using each mAb either as a capture or gold-labeled antibody. In this study, nine antibodies selected on the basis of their previous performance were evaluated (AP406, AP430, AP3202, AP267, AP476, AP2573, AP3659, AP3808, and KAP142).

#### 4.11.2. Preparation of Colloidal Gold Labeled Abrin Antibodies

Colloidal gold was prepared as previously described [80]. In total, 2 mL of the colloidal gold solution was centrifuged for 15 min at 15,000× *g*, and the pellet was suspended in 1.6 mL of water. Then, 200 µL of a 100 µg/mL solution of each mAb in 0.02 M phosphate buffer at pH 7.4 was added to the colloidal/gold and incubated for 1 h at 20 °C, leading to the ionic adsorption of the mAbs on the surface of the gold particles. Then, 200 µL of phosphate buffer 20 mM at pH 7.4 containing BSA 1% (*w*/*v*) was added, and the mixture was centrifuged for 15 min at 15,000× *g*. The supernatant was discarded and the pellet suspended in 1 mL phosphate buffer 2 mM at pH 7.4 with BSA 0.1% (*w*/*v*), sonicated a few seconds and centrifuged for 15 min at 15,000× *g*. The supernatant was discarded and the pellet suspended in 500 µL of phosphate buffer 2 mM at pH 7.4 with BSA 0.1% (*w*/*v*) and stored at 4 °C in the dark.

#### 4.11.3. Selection and Assessment of the Best Pairs of Antibodies

The spotting method was performed as previously described [81]. In total, 1 µL of each mAb (100 µg/mL in 50 mM phosphate buffer pH 7.4) was applied on the strips and allowed to dry. Then, 100 µL of an abrin solution (30 ng/mL in 100 mM potassium phosphate at pH 7.4 with 0.1% (*w*/*v*) BSA, 150 mM NaCl, 0.01% (*w*/*v*) NaN_3_, 0.5% (*v*/*v*) Tween 20) and 10 µL of colloidal gold labeled mAb were delivered in microtiter plate wells (Greiner, Les Ulis, France). After a 5 min reaction, the strips were immersed into the solution, and results were read out after 30 min of migration.

For the assessment of superior mAb pairs, a conventional strip format was used. The strips (0.5 cm in width and 4.5 cm in length) were composed of three parts: (i) a sample pad (Standard 14; Whatman) (0.5 cm in length), (ii) a nitrocellulose membrane (Prima 40, Cytiva, Velizy-Villacoublay, France) (2.5 cm in length) and (iii) an absorption pad (Cellulose grade 470; Whatman) (1.5 cm in length), all attached to a backing card. The detection zone contained immobilized goat anti-mouse antibodies as a control line and an anti-abrin mAb as a test line (1 mg/mL in 50 mM sodium phosphate buffer; pH 7.4) dispensed at 1 µL/cm using an automatic dispenser (Biojet XYZ 3050; BioDot, Norton, UK). After drying for 30 min at 37 °C in an air oven, the membrane was incubated with a blocking solution (10 mM sodium phosphate pH 7.4, 150 mM NaCl containing 0.5% (*w*/*v*) BSA) for 30 min at RT. The membrane was washed three times with deionized water, incubated for 30 min at RT in a preserving solution (10 mM sodium phosphate pH 7.4, 150 mM NaCl containing 0.1% (*v*/*v*) Tween 20 and 7.5% (*w*/*v*) glucose) and then dried for 30 min at 37 °C in an air oven. After the absorption pad and the sample pad were fixed to the top and the bottom of the membrane, respectively, the card was cut into strips 5 mm in width using an automatic programmable cutter (CM4000 Guillotine cutting system; BioDot, Norton, UK).

Dilutions of abrin (40, 20, 10, 5, 2.5, 0.1, and 0 ng/mL) were performed in an extraction buffer (Tris 100 mM, pH 8, NaCl 0.15 M, BSA 0.1% (*w*/*v*), 0.5% (*v*/*v*) Tween 20, 1% (*w*/*v*) CHAPS). In total, 100 µL of this solution was incubated for 5 min with 10 µL of conjugate before dripping the strip. Results were read out after 30 min of migration time.

#### 4.11.4. Evaluation of the LFA

For the evaluation of the limit of detection (LOD), industrially made strips (NG Biotech, Guipry, France) were used. In total, 50 µL of diluted abrin (200, 100, 50, 25, 12.5, 6, and 0 ng/mL) in 0.1% BSA/PBS was further diluted to a ratio of 1:10 in extraction buffer (100 mM Tris pH 8, 150 mM NaCl, 0.1% BSA (*w*/*v*), 0.5% (*v*/*v*) Tween 20, and 1% (*w*/*v*) CHAPS) according to the manufacturer’s instructions. Then, 100 µL of this solution was applied to the sample well of the abrin LFA. Analysis was done with the naked eye after 30 min.

To assess matrix compatibility, the buffer (0.1% BSA/PBS), semi-skimmed milk and 10% cat feces suspension were spiked with 500 and 50 ng/mL abrin and incubated for 30 min under rotation at 4 °C. After centrifugation, 50 µL was mixed with 450 µL extraction buffer (1:10 dilution, according to manufacturer’s instructions) and 100 µL of this solution was applied to the sample well of the abrin LFA. Analysis was done with the naked eye after 30 min. 

One part of reconstituted soil sample was mixed with nine parts of extraction buffer and spiked with 50 or 5 ng/mL abrin. After 30 min of incubation under rotation at 4 °C and centrifugation, 100 µL was directly applied to the sample well of the abrin LFA. Analysis was done with the naked eye after 30 min.

### 4.12. Mass Spectrometry

#### 4.12.1. Peptide Mass Fingerprinting by MALDI-TOF-MS

For purity control, the purified abrin and APA preparations were diluted to approximately 600 ng in 25 µL of trypsin digest buffer (40 mM NH_4_HCO_3_ containing 9% (*v*/*v*) acetonitrile). Reduction was performed with 1.5 µL of 400 mM dithiothreitol for 10 min at 95 °C under shaking. Alkylation was carried out using 3.0 µL of 500 mM iodoacetamide at 37 °C for 30 min in the dark. Reduced and alkylated samples were digested with 15 µL of trypsin (0.02 ng/µL, proteomics grade; Sigma-Aldrich, Taufkirchen, Germany) at 37 °C overnight. Reaction was stopped with 4 µL of 0.1% trifluoroacetic acid. Digested peptides were further desalted and concentrated with ZipTip C18 resin (Merck Millipore, Darmstadt, Germany), which was carried out according to the manufacturer’s instructions. Sample analysis was done utilizing an autoflex speed MALDI-TOF/TOF mass spectrometer (Bruker Daltonics, Bremen, Germany) with a polished steel MTP 384 target plate (Bruker Daltonics, Bremen, Germany). One microliter of sample was mixed with 1 µL of ɑ-cyano-4-hydroxycinnamic acid (12 mg/mL; Bruker Daltonics, Bremen, Germany), and 1 µL of the mixture was deposited on the target to let it dry. For matrix suppression, deflection was set to 600, and mass spectra were acquired over the mass range of 600–4500. External calibration was performed with peptide calibration standard II (Bruker Daltonics, Bremen, Germany). Spectra were processed by flexAnalysis 2.4 (Bruker Daltonics, Bremen, Germany) and MASCOT server 2.4 software (Matrix Science Ltd., London, UK).

#### 4.12.2. LC-ESI-MS/MS with Immuno-Affinity-Enrichment

Monoclonal antibodies directed against abrin (namely AP430, AP3659, AP3808) or APA (AP476) were immobilized on M-280 tosylactivated magnetic Dynabeads^®^ (Life Technologies, Oslo Norway) as described by Kull et al. [82]. Briefly, resuspended Dynabeads^®^ (250 μL) were washed twice with 800 μL of buffer A (0.1 M sodium phosphate buffer, pH 7.4), mixed separately with 150 μg of each mAb and incubated at 37 °C overnight, under rotation. The reaction was stopped by washing the beads twice with 800 μL of buffer B (0.1% BSA/PBS) for 5 min each at 4 °C, resuspending in 800 μL of buffer C (0.2 M Tris containing 0.1% BSA, pH 8.5) and incubating at 37 °C for 4 h. Beads were washed with 800 μL of buffer B and stored in 500 μL of buffer B at 4 °C.

For the immuno-enrichment of abrin and APA, an antibody–bead mix containing 8 µL of AP430–Dynabeads, 4 µL of AP3808–Dynabeads, 4 µL of AP3659–Dynabeads and 8 µL of AP476–Dynabeads was added to 50 µL abrin (~75 µg abrin) or APA (~85 µg APA) in a KingFisher^TM^ deep well plate (Thermo Fisher Scientific, Bremen, Germany). The sample was diluted with 400 µL 1× phosphate buffered saline with 0.05% (*v/v*) Tween 20 (PBST) as well as with 50 µL of 10× PBST. The deep well plate was placed in a KingFisher flex purification system (Thermo Fisher Scientific, Bremen, Germany) for automated bead shaking (2 h) and washing, which included two washes with 1 mL each of PBST followed by one wash with 1 mL of PBS. Beads were eluted into 1 mL of water, removed from the KingFisher flex system and separated manually on a DynaMag-2 magnet (Life Technologies, Oslo, Norway). Supernatants were discarded and the toxin was eluted with 25 µL of 0.1% (*v/v*) Trifluoroacetic acid (TFA, Merck, Darmstadt, Germany) in Ultra LC-MS-grade water (Carl Roth, Karlsruhe, Germany) for 10 min. Supernatants were transferred to a fresh LoBind Eppendorf tube (Hamburg, Germany) and neutralized with 7 μL of 400 mM NH_4_HCO_3_. Dithiosulfide bond reduction and alkylation was performed by adding 1.5 µL of 400 mM dithiothreitol (DTT, Sigma-Aldrich, Munich, Germany) and submitting the mixture to 10 min of shaking at 95 °C. After cooling to RT, 3 μL of 500 mM 2-iodoacetamide (Sigma-Aldrich, Munich, Germany) was added and incubated for 30 min at 37 °C. Protein digestion was achieved by the addition of 5 μL of 20 μg/mL proteomics grade trypsin solution (Sigma-Aldrich, Munich, Germany) followed by o/n incubation at 37 °C. Digestion reaction was stopped by adding 4 μL of 10% trifluoroacetic acid (TFA, Sigma-Aldrich, Munich, Germany). The sample was desalted with ZipTip C18 (Merck, Darmstadt, Germany) according to the manufacturer’s protocol. ZipTip eluate was dried in a speedvac concentrator (Thermo Fisher Scientific, Bremen, Germany) and resuspended in 15 μL of 0.1% formic acid (Thermo Scientific, Bremen, Germany). The concentration of digested peptides was determined by absorbance measurement at 280 nm in a NanoPhotometer (Thermo Fisher Scientific, Bremen, Germany). Peptides were analyzed on a nanoLC (EASY-nanoLC 1200, Thermo Fisher Scientific, Bremen, Germany) coupled online to an Orbitrap mass spectrometer (Q Exactive^TM^ Plus or Q Exactive^TM^ HF, Thermo Fisher Scientific, Bremen). Peptide solution (5 µL) was loaded on an Acclaim^TM^ PepMap^TM^ trap column (20 mm × 75 μm i.d., 100 Å C18, 3 μm; Thermo Fisher Scientific, Bremen, Germany) at a flow rate of 3 μL/min, followed by peptide separation on a 200 cm μPAC column (PharmaFluidics, Ghent, Belgium) using a linear 60 min gradient of 4% to 43% acetonitrile in 0.1% of formic acid at a 300 nL/min flow rate. The temperature of the LC column was set to 50 °C. The mass spectrometer was operated in a data-dependent acquisition mode and the following settings were applied: full scan spectra (MS^1^) were recorded with a scan resolution of 70,000 in a scan range of 300 to 1650 *m*/*z*. The MS^1^ automatic gain control (AGC) target value was set to 5 × 10^5^ with a maximum injection time of 20 ms. Fragment spectra (MS^2^) were obtained by higher-energy c-trap dissociation (HCD) with a normalized collision energy (NCE) of 25% for up to the 12 most intense 2^+^ to 5^+^ charged ions. MS^2^ scan resolution was 17,500 at 200 *m*/*z*. MS^2^ AGC target value was set to 1 × 10^5^ with a maximum injection time of 50 ms and an isolation window of 1.5 *m*/*z*. The minimum AGC target value was set to 1 × 10^4^ and a dynamic exclusion of 30 s within a 10 ppm window. Peptides were ionized using electrospray with a stainless-steel emitter, I.D. 30 μm, (Proxeon, Odense, Denmark) at a spray voltage of 2.0 kV and a heated capillary temperature of 275 °C. Mass data were processed by Proteome Discover software (Thermo Fisher Scientific, Bremen, Germany) as well as MASCOT server 2.4 software (Matrix Science Ltd., London, UK).

## Figures and Tables

**Table 1:** Amino acid-sequence identity of abrin compared to *Abrus precatorius* agglutinin and the related proteins ricin and RCA120 from *Ricinus communis* *.

	Abrin	APA	Ricin	RCA120
**Abrin**	-	74.8	48.8	44.6
**APA**	74.8	-	44.9	42.5
**Ricin**	48.8	44.9	-	89.6
**RCA120**	44.6	42.5	89.6	-

* Amino acid-sequence identity in percent [%]. The following protein sequences from the UniProt database were used: abrin-a, ABRA_ABRPR/P11140; *Abrus precatorius* agglutinin (APA), AGGL_ABRPR/Q9M6E9; ricin, RICI_RICCO/P02879; and *Ricinus communis* agglutinin (RCA120), AGGL_RICCO/P06750. Alignment by MUSCLE Alignment (3.8.425) with a maximum of eight iterations.

**Table 2:** Characteristics of monoclonal antibodies against abrin or APA. Antibodies indicated in bold were used for setting up ELISA, lateral flow assay (LFA) and/or mass spectroscopy (MS) analyses (this publication and [42,43]).

Antibody	Isotype	Affinity *K_D_* [M]		Specificity *	
		Abrin	APA	Abrin ^§^	APA ^§^
AP12	IgG1	n. b.	n. b.	+	–
AP54	IgG1	n. b.	n. b.	+++	0
AP69	IgG1	n. b.	n. b.	+++ (B)	0
AP87	IgG2a	n. b.	n. b.	+++	+++
AP188	IgG1	n. b.	n. b.	+++ (A)	0
AP267	IgG2a	1.4 × 10^−7^	6.8 × 10^−10^	++	+++
AP406	IgG1	9.1 × 10^−9^	5.0 × 10^−8^	+++	0
**AP430**	**IgG2a**	**1.5 × 10^−9^**	**4.3 × 10^−8^**	**+++ (B)**	**+**
AP464	IgG2a	n. b.	n. b.	+++ (B)	+++
**AP476**	**IgG2a**	**1.8 × 10^−7^**	**<10^−10^ #**	**++**	**+++**
AP708	IgG2a	n. b.	n. b.	+++ (B)	+++
**AP2573**	**IgG1**	**5.9 × 10^−8^**	**<10^−10^ #**	**0**	**+++**
**AP3202**	**IgG1**	**3.3 × 10^−9^**	**n. b.**	**+++ (A)**	**0**
**AP3659**	**IgG2a**	**1.1 × 10^−8^**	**n. b.**	**+++**	**0**
**AP3808**	**IgG2a**	**1.1 × 10^−8^**	**n. b.**	**+++**	**0**

* Specificity is shown as derived from indirect ELISA, Western blots and, where possible, by SPR experiments. # High affinity binding, dissociation out of measurement range of the instrument; ^§^ Based on A_[450−620 nm]_ ELISA readings (Figure 1) **–**: <0.2; 0: <0.5; **+** <1; ++: <2; +++: ≥2; n.b.: no binding in the surface plasmon resonance (SPR) setting applied (Supplementary Figure S4). (A) Epitope of the antibody localized on the abrin A-chain; (B) epitope of the antibody localized on the abrin B-chain.

**Table 3:** Key features of the abrin-specific ELISA as determined in a validation study.

Parameter *	Abrin-Specific ELISA
EC_50_ (pg/mL)	372 ± 60 pg/mL
LOD (pg/mL)	22 ± 6 pg/mL
LLOQ	109 ± 20 pg/mL
ULOQ	1270 ± 210 pg/mL
CV_intra_ (EC_50_)	8%
CV_inter_ (EC_50_)	12%

* EC_50_, half maximal effective concentration; LOD, the limit of detection; LLOQ and ULOQ, lower and upper limits of quantification; CV_intra_ and CV_inter_, intra-assay and inter-assay coefficients of vari-ation measured at EC_50_.

**Table 4:** Performance of the mAbs generated in this work in various applications.

Antibody Short Name	Application of Antibodies #				
	Western Blot	SPR	Sandwich ELISA *	LFA *	Immuno-Enrichment followed by MS Analysis * ^§^
AP12	–	–			
AP54	X	–			
AP69	X	–			
AP87	X	–			
AP188	X	–			
AP267	X	X			
AP406	–	X			
**AP430**	**X**	**X**	**X**	**X**	**X**
AP464	X	–			
**AP476**	**X**	**X**	**X**		**X**
AP708	X	–			
**AP2573**	**–**	**X**	**X**		
**AP3202**	**X**	**X**	**X**		
**AP3659**	**–**	**X**			**X**
**AP3808**	**–**	**X**		**X**	**X**

* selected antibodies with superior performance for the indicated method (bold); # X: good performance; –: no or poor binding; § immuno-enrichment for MS as described in this publication and in Hansbauer et al. [42] and Livet et al. [43].

## Data Availability

Data is contained within the manuscript or the supplementary information. The data presented in this study is available in Worbs, S.; Kampa, B.; Skiba, M.; Hansbauer, E.-M.; Stern, D.; Volland, H.; Becher, F.; Simon, S.; Dorner, M.B.; Dorner, B.G. Differentiation, Quantification and Identification of Abrin and *Abrus precatorius* Agglutinin. *Toxins* **2021**, *13*, 284. https://doi.org/10.3390/toxins13040284.

## Supplementary Materials

The following are available online at https://www.mdpi.com/article/10.3390/toxins13040284/s1, Figure S1: Purified abrin and *A. precatorius* agglutinin (APA) analyzed by SDS-PAGE and Coomassie staining. Figure S2: Purified abrin and *A. precatorius* agglutinin (APA) analyzed by MALDI-TOF MS. Figure S3: Detection of purified abrin, APA or ricin by Western blot using the monoclonal antibodies generated in this work. Figure S4: Binding kinetics of the newly generated monoclonal antibodies to abrin and APA. Figure S5: Binding specificity of selected monoclonal antibodies targeting the A or B-chain of purified abrin-a. Figure S6: Protein sequence coverage of proteins identified in the purified abrin preparation. Figure S7: Protein sequence coverage of proteins identified in the purified APA preparation.
