# Supplementary material for: Differentiation, Quantification and Identification of Abrin and Abrus precatorius Agglutinin
Source: Toxins (Basel). 2021 Apr 18;13(4):284. doi: 10.3390/toxins13040284 (PMC8073929; doi:10.3390/toxins13040284)
Supplement: Supplementary file 1 [file toxins-13-00284-s001.zip › toxins-1163523-supplementary final.pdf]

Supplementary Materials: Differentiation, Quantification and Identification of Abrin and *Abrus precatorius* Agglutinin

Sylvia Worbs, Bettina Kampa, Martin Skiba, Eva-Maria Hansbauer, Daniel Stern, Hervé Volland, François Becher, Stéphanie Simon, Martin B. Dorner and Brigitte G. Dorner

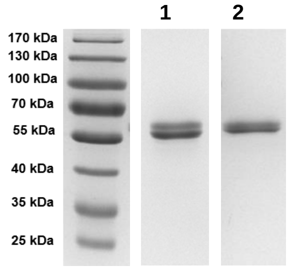

Figure S1. Purified abrin and *A. precatorius* agglutinin (APA) analyzed by SDS-PAGE and Coomassie staining. Two µg of abrin (lane 1) or APA (lane 2) each were separated on 12% gels by SDS-PAGE under non-reducing conditions followed by staining with colloidal Coomasssie Brilliant Blue over night.

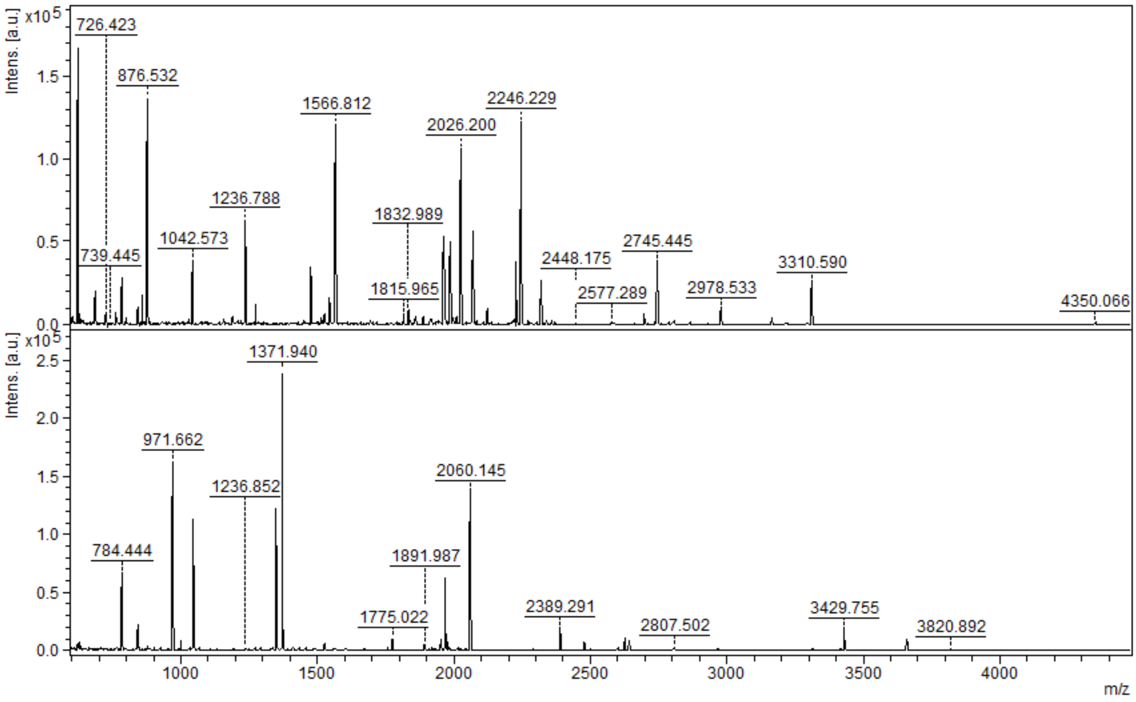

Figure S2. Purified abrin and *A. precatorius* agglutinin (APA) analyzed by MALDI-TOF MS. Overview of peptide mass fingerprinting (PMF) MALDI-TOF MS spectra of approximately 600 ng of purified abrin (top) or APA (bottom) after reduction, alkylation and tryptic *in-solution* digest. Peaks are labelled with the corresponding *m*/*z* value.

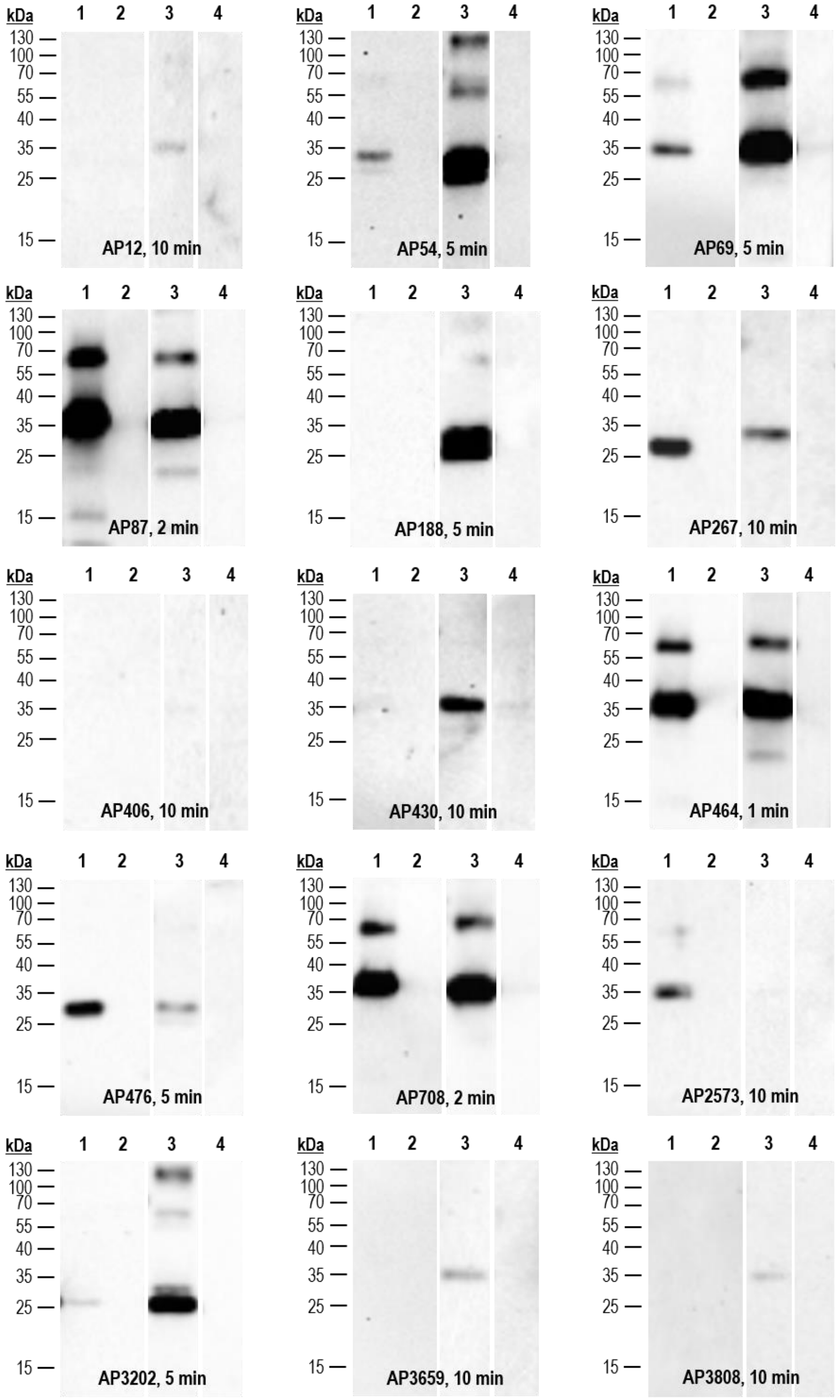

Figure S3. Detection of purified abrin, APA or ricin by Western blot using the monoclonal antibodies generated in this work. Purified APA (lane 1), BSA (as negative control, lane 2), abrin (lane 3) and ricin (lane 4), each 100 ng, were separated by SDS-PAGE under reducing conditions and blotted onto PVDF membranes. For detection the indicated monoclonal antibodies were used. For development a biotin-coupled anti-mouse antibody followed by streptavidin alkaline phosphatase and CDP star as

chemiluminescent substrate were used. Exposure time of the blots was 1–10 min as indicated. As positive control the polyclonal rabbit antibody KAP142 was used in Western blotting which detected both purified *Abrus* lectins (abrin and APA, main reactivity) as well as ricin (lower signals, data not shown).

(a)

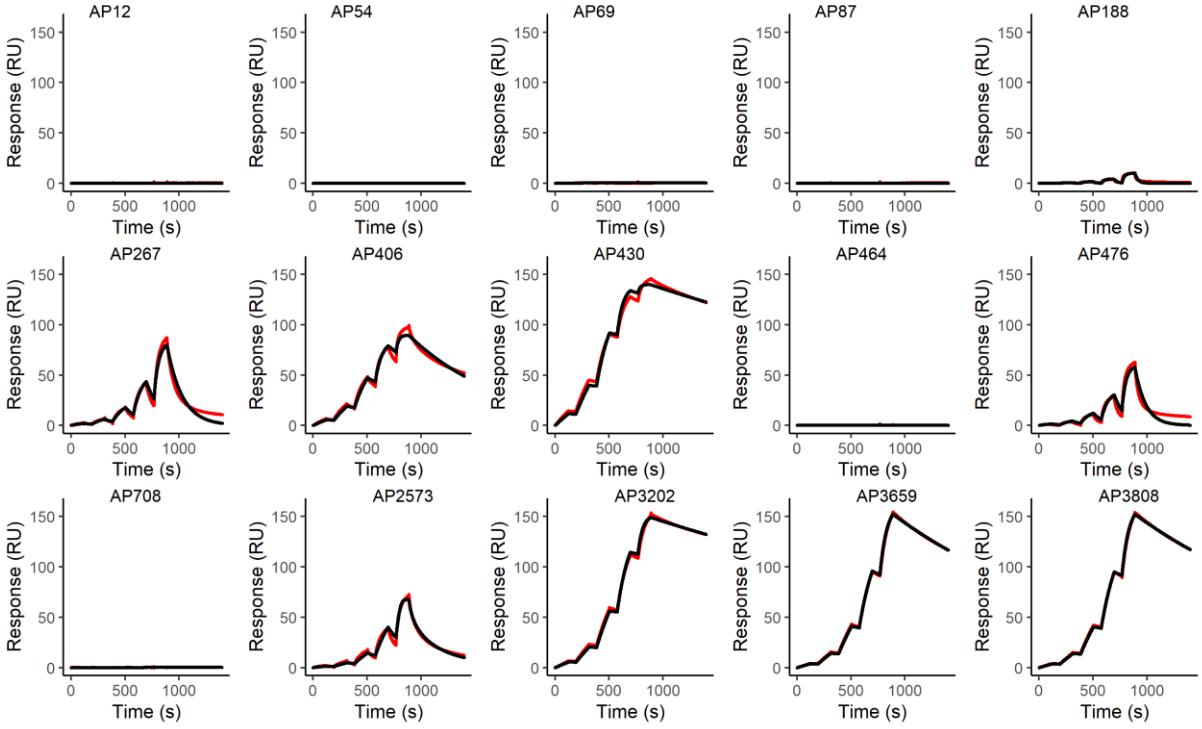

(b)

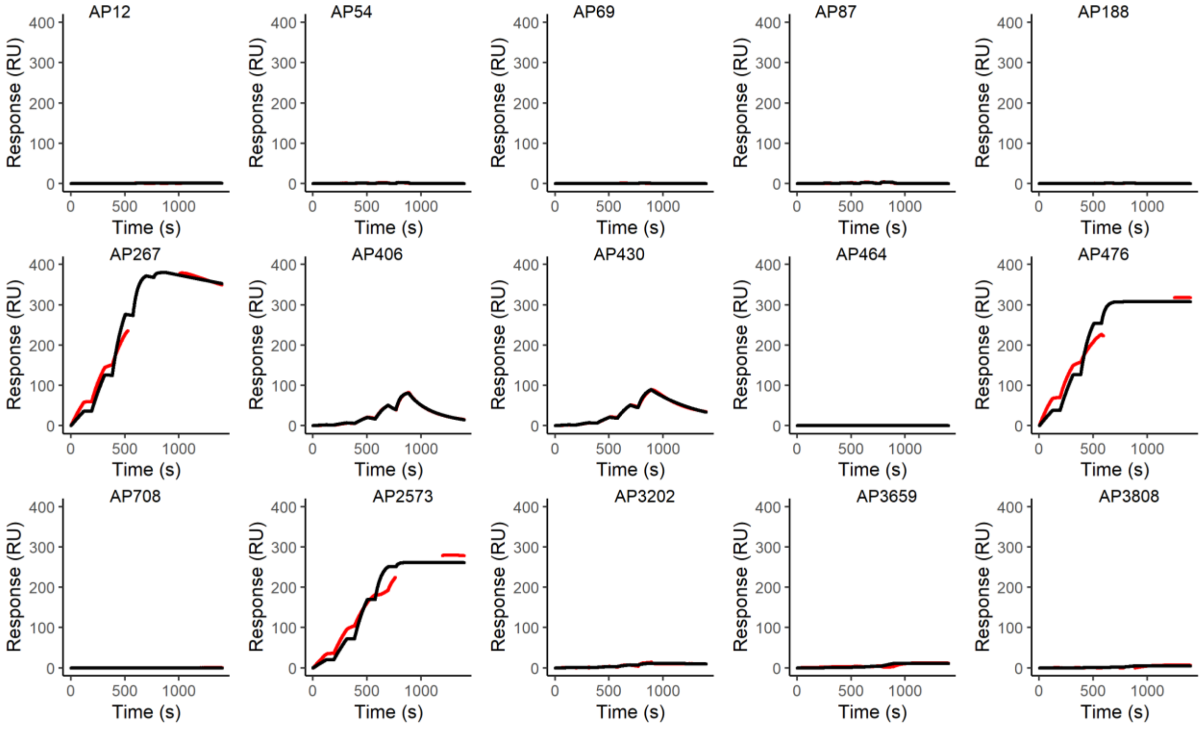

Figure S4. Binding kinetics of the newly generated monoclonal antibodies to abrin and APA. Shown are binding responses (in resonance units RU) of double referenced binding curves (red lines) overlaid with fitting curves (black lines) from a 1:1 binding model for single cycle kinetic measurements of the indicated mAbs binding to (**a**) abrin or (**b**) APA. Five increasing concentrations of abrin or APA were

injected consecutively for 120 s before buffer was injected for 600 s after injection of the highest concentration (333.33 nM corresponding to 20 µg/mL abrin or 40 µg/mL APA).

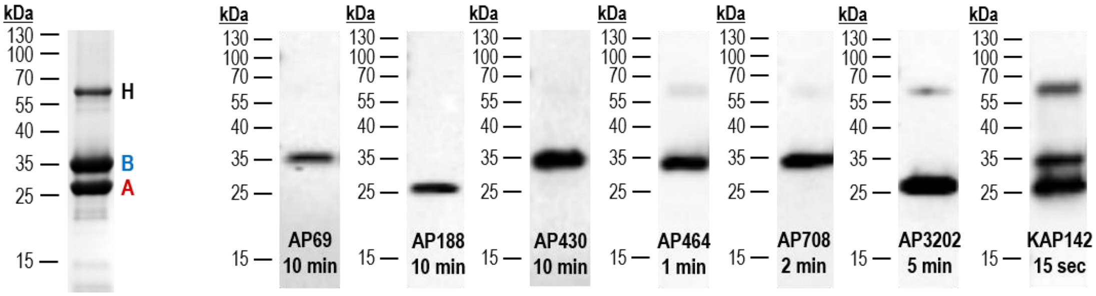

(**a**) (**b**)

Figure S5. Binding specificity of selected monoclonal antibodies targeting the A- or B-chain of purified abrin-a. (**a**) 15 µg of purified abrin-a were separated by SDS-PAGE under reducing conditions followed by staining with colloidal Coomasssie Brilliant Blue overnight. H: band corresponding to non-reduced abrin-a holotoxin; B: band corresponding to B-chain of abrin-a; A: band corresponding to A-chain of abrin-a [1,2]. The assignments of the A- and B-chains were confirmed by in-gel digest followed by MALDI-TOF MS analysis. (**b**) Purified abrin-a was analyzed by Western blot using several monoclonal antibodies generated in this work. 100 ng of abrin-a were separated by SDS-PAGE under reducing conditions and blotted onto PVDF membranes. For detection the indicated monoclonal antibodies were used. As a positive control the polyclonal rabbit antibody KAP142 was used to detect both sub-units of purified abrin-a (A- and B-chain). For development a biotin-coupled anti-mouse antibody (for the detection of monoclonal antibodies) or a biotin-coupled anti-rabbit antibody (for the detection of KAP142) followed by streptavidin alkaline phosphatase and CDP star as chemiluminescent substrate was used. Exposure time of the blots was 15 s to 10 min as indicated.

P11140, **Abrin-a**; Protein sequence coverage: 60%

1 QDRPIK**FSTE GATSQSYKQF IEALRERLRG GLIHDIPVLP DPTTLQER**NR

51 YITVELSNSD TESIEVGIDV TNAYVVAYR**A GTQSYFLRDA PSSASDYLFT**

101 **GTDQHSLPFY GTYGDLERWA HQSRQQIPLG LQALTHGISF FRSGGNDNEE**

151 **KARTLIVIIQ MVAEAARFRY ISNRVR**VSIQ TGTAFQPDAA MISLENNWDN

201 LSR**GVQESVQ DTFPNQVTLT NIR**NEPVIVD SLSHPTVAVL ALMLFVCNPP

251 NANQSPLLIR **SIVEKSKICS SRYEPTVRIG GRDGMCVDVY DNGYHNGNRI**

301 **IMWKCKDRLE ENQLWTLKSD KTIR**SNGKCL TTYGYAPGSY VMIYDCTSAV

351 AEATYWEIWD NGTIINPK**SA LVLSAESSSM GGTLTVQTNE YLMR**QGWRTG

401 NNTSPFVTSI SGYSDLCMQA QGSNVWMADC DSNK**KEQQWA LYTDGSIRSV**

451 **QNTNNCLTSK DHKQGSTILL MGCSNGWASQ R**WVFK**NDGSI YSLYDDMVMD**

501 **VKGSDPSLK**Q IILWPYTGKP NQIWLTLF

Q06077, **Abrin-b**; Protein sequence coverage: 60%

1 QDQVIK**FTTE GATSQSYKQF IEALRQRLTG GLIHGIPVLP DPTTLQERNR**

51 YISVELSNSD TESIEAGIDV SNAYVVAYR**A GNRSYFLRDA PTSASRYLFT**

101 **GTQQYSLRFN GSYIDLER**LA RQTR**QQIPLG LQALRHAISF LQSGTDDQEI**

151 **ARTLIVIIQM ASEAARYRFI SYRVGVSIR**T NTAFQPDAAM ISLENNWDNL

201 SGGVQQSVQD TFPNAVTLRS VNNQPVIVDS LTHQSVAVLA LMLFVCNPPN

251 ANQSPLLIR**S IVEKSKICSS RYEPTVRIGG RNGMCVDVYD DGYHNGNRII**

301 **AWKCKDRLEE NQLWTLKSDK TIR**SNGKCLT TEGYAPGNYV MIYDCTSAVA

351 EATYWEIWDN GTIINPK**SAL VLSAESSSMG GTLTVQTNEY LMR**QGWRTGN

401 NTSPFVTSIS GYSDLCMQAQ GSNVWLAYCD NNK**KEQQWAL YTDGSIRSVQ**

451 **NTNNCLTSKD HKQGSPIVLM ACSNGWASQR** WLFR**NDGSIY NLHDDMVMDV**

501 **KRSDPSLKEI ILHPYHGKPN QIWLTLF**

P28590, **Abrin-c**; Protein sequence coverage: 55%

1 MDKTLKLLIL CLAWTCSFSA LRCAARTYPP VATNQDQVIK **FTTEGATSQS**

51 **YKQFIEALR**Q R**LTGGLIHDI PVLPDPTTVE ERNRYITVEL SNSER**ESIEV

101 GIDVTNAYVV AYR**AGSQSYF LRDAPASAST YLFPGTQR**YS LR**FDGSYGDL**

151 **ERWAHQTR**EE ISLGLQALTH AISFLR**SGAS NDEEKARTLI VIIQMASEAA**

201 **RYRYISNRVG VSIR**TGTAFQ PDPAMLSLEN NWDNLSGGVQ QSVQDTFPNN

251 VILSSINRQP VVVDSLSHPT VAVLALMLFV CNPPNANQSP LLIR**SIVEES**

301 **KICSSRYEPT VRIGGRDGMC VDVYDDGYHN GNRIIAWKCK DRLEENQLWT**

351 **LKSDKTIR**SN GKCLTTEGYA PGNYVMIYDC TSAVAEATYW EIWDNGTIIN

401 PK**SALVLSAE SSSMGGTLTV QTNEYLMR**QG WRTGNNTSPF VTSISGYSDL

451 CMQAQGSNVW LADCDNNK**KE QQWALYTDGS IRSVQNTNNC LTSKDHKQGS**

501 **PIVLMACSNG WASQR**WLFK**N DGSIYNLHDD MVMDVKRSDP SLKEIILHPY**
551 **HGKPNQIWLT LF**

Q06076, **Abrin-d**; Protein sequence coverage: 53%
1 QDQVIK**FTTE GATSQSYKQF IEALR**QR**LTG GLIHDIPVLP DPTTVEERNR**
51 **YITVELSNSE R**ESIEVGIDV TNAYVVAYR**A GSQSYFLRDA PASASTYLFP**
101 **GTQR**YSLR**FD GSYGDLERWA HQTR**EEISLG LQALTHAISF LR**SGASNDEE**
151 **KARTLIVIIQ MASEAAR**YRC ISNR**VGVSIR** TGTAFQPDPA MLSLENNWDN
201 LSGGVQQSVQ DAFPNNVILS SINRQPVVVD SLSHPTVAVL ALMLFVCNPP
251 NANQSPLLIR **SIVEESKICS SRYEPTVRIG GRDGMCVDVY DDGYHNGNRI**
301 **IAWKCKDRLE ENQLWTLK**SD LTIRSNGKCL TTEGYAPGNY VMIYDCTSAV
351 AEATYWEIWD NGTIINPK**SA LVLSAESSSM GGTLTVQTNE YLMR**QGWRTG
401 NNTSPFVTSI SGYSDLCMQA QGSNVWLADC DNNK**KEQQWA LYTDGSIRSV**
451 **QNTNNCLTSK DHKQGSPIVL MACSNGWASQ R**WLFK**NDGSI YSLYDDMVMD**
501 **VKGSDPSLK**Q IILWPYTGKP NQIWLTLF

Q9M6E9, **Agglutinin-1**; Protein sequence coverage: 39%
1 MKFETTKNKL HGNAYYQAQF QDPIK**FTTGS ATPASYNQFI DALRERLTGG**
51 **LIYGIPVLR**D PSTVEKPNQY VTVELSYSDT VSIQLGIDLT NAYVVAYR**AG**
101 **SESFFFR**NAP ASASTYLFTG TQQYSLPFDG NYDDLEK**WAH QSRQRISLGL**
151 **EALR**QGIKFL R**SGASDDEEI ARTLIVIIQM VAEAARFRYV SKLVVISLSN**
201 **RAAFQPDPSM LSLENTWEPL SRAVQHTVQD TFPQNVTLIN VR**QERVVVSS
251 LSHPSVSALA LMLFVCNPLN ATQSPLLIRS VVEQSK**ICSS HYEPTVR**IGG
301 RDGLCVDVSD NAYNNGNPII LWK**CKDQLEV NQLWTLKSDK TIR**SKGKCLT
351 TYGYAPGNYV MIYDCSSAVA EATYWDIWDN GTIINPK**SGL VLSAESSSMG**
401 **GTLTVQK**NDY RMRQGWRTGN DTSPFVTSIA GFFKLCMEAH GNSMWLDVCD
451 ITKEEQQWAV YPDGSIRPVQ NTNNCLTCEE HK**QGATIVMM GCSNAWASQR**
501 WVFKSDGTIY NLYDDMVMDV K**SSDPSLK**QI ILWPYTGNAN QMWATLF

Figure S6. Protein sequence coverage of proteins identified in the purified abrin preparation. Approximately 75 µg of the purified abrin preparation used in this work were subjected to immunoaffinity enrichment using a mixture of four mAbs, namely AP430, AP3808, AP3659 and AP476, coupled to magnetic Dynabeads, followed by protein reducing, alkylation, tryptic digest and non-targeting LC-ESI-MS/MS analysis. From top to bottom, sequences of identified abrin isoforms (UniProt P11140, UniProt Q06077, UniProt P28590, UniProt Q06076) and *Abrus precatorius* Agglutinin APA; UniProt Q9M6E9) are shown after MASCOT server search against a self-assembled UniProt/NCBI database containing all abrin isoforms and *Abrus precatorius* agglutinin as well as an NCBI database containing all *Abrus precatorius* proteins. Asparagine (N) highlighted in turquoise represents potential N-linked glycosylation sites. The linker peptide sequence between the two chains of each abrin isoform is underlined. Amino acids highlighted in red were experimentally identified. Amino acids in position 1–34 in abrin-c or in position 1–20 in APA represent the signal peptide

(highlighted in grey). CAVE: Unambigous identification of abrin-c in the presence of the other three isoforms (abrin-a, -b and -d) is not possible with our current LC-MS/MS setup-up so that no proteotypic peptides for abrin-c can be detected.

Q9M6E9, **Agglutinin-1**; Protein sequence coverage: 52%

1 MKFETTKNKL HGNAYYQAQF QDPIK**FTTGS ATPASYNQFI DALRERLTGG**
51 **LIYGIPVLR**D PSTVEKPNQY VTVELSYSDT VSIQLGIDLT NAYVVAYR**AG**
101 **SESFFFRNAP ASASTYLFTG TQQYSLPFDG NYDDLEKWAH QSRQRISLGL**
151 **EALRQGIKFL RSGASDDEEI ARTLIVIIQM VAEAARFRYV SKLVVISLSN**
201 **RAAFQPDPSM LSLENTWEPL SR**AVQHTVQD TFPQNVTLIN VRQERVVVSS
251 LSHPSVSALA LMLFVCNPLN ATQSPLLIR**S VVEQSKICSS HYEPTVRIGG**
301 **RDGLCVDVSD NAYNNGNPII LWKCKDQLEV NQLWTLKSDK** TIRSKGKCLT
351 TYGYAPGNYV MIYDCSSAVA EATYWDIWDN GTIINPK**SGL VLSAESSSMG**
401 **GTLTVQKNDY R**MRQGWRTGN DTSPFVTSIA GFFKLCMEAH GNSMWLDVCD
451 ITKEEQQWAV YPDGSIRPVQ NTNNCLTCEE HK**QGATIVMM GCSNAWASQR**
501 WVFK**SDGTIY NLYDDMVMDV KSSDPSLK**QI ILWPYTGNAN QMWATLF

P11140, **Abrin-a**; Protein sequence coverage: 53%

1 QDRPIK**FSTE GATSQSYKQF IEALRERLRG GLIHDIPVLP DPTTLQERNR**
51 YITVELSNSD TESIEVGIDV TNAYVVAYR**A GTQSYFLRDA PSSASDYLFT**
101 **GTDQHSLPFY GTYGDLERWA HQSRQQIPLG LQALTHGISF FR**SGGNDNEE
151 KAR**TLIVIIQ MVAEAARFRY ISNRVR**VSIQ TGTAFQPDAA MISLENNWDN
201 LSR**GVQESVQ DTFPNQVTLT NIR**NEPVIVD SLSHPTVAVL ALMLFVCNPP
251 NANQSPLLIR **SIVEKSKICS SRYEPTVRIG GRDGMCVDVY DNGYHNGNRI**
301 **IMWKCKDRLE ENQLWTLKSD KTIR**SNGKCL TTYGYAPGSY VMIYDCTSAV
351 AEATYWEIWD NGTIINPKSA LVLSAESSSM GGTLTVQTNE YLMRQGWRTG
401 NNTSPFVTSI SGYSDLCMQA QGSNVWMADC DSNK**KEQQWA LYTDGSIRSV**
451 **QNTNNCLTSK DHKQGSTILL MGCSNGWASQ R**WVFK**NDGSI YSLYDDMVMD**
501 **VKGSDPSLK**Q IILWPYTGKP NQIWLTLF

Q06077, **Abrin-b**; Protein sequence coverage: 54%

1 QDQVIK**FTTE GATSQSYKQF IEALRQRLTG GLIHGIPVLP DPTTLQERNR**
51 YISVELSNSD TESIEAGIDV SNAYVVAYRA GNR**SYFLRDA PTSASRYLFT**
101 **GTQQYSLRFN GSYIDLER**LA RQTR**QQIPLG LQALRHAISF LQSGTDDQEI**
151 **ARTLIVIIQM ASEAARYRFI SYRVGVSIR**T NTAFQPDAAM ISLENNWDNL
201 SGGVQQSVQD TFPNAVTLRS VNNQPVIVDS LTHQSVAVLA LMLFVCNPPN
251 ANQSPLLIR**S IVEKSKICSS RYEPTVRIGG RNGMCVDVYD DGYHNGNRII**
301 **AWKCKDRLEE NQLWTLKSDK TIR**SNGKCLT TEGYAPGNYV MIYDCTSAVA
351 EATYWEIWDN GTIINPKSAL VLSAESSSMG GTLTVQTNEY LMRQGWRTGN
401 NTSPFVTSIS GYSDLCMQAQ GSNVWLAYCD NNK**KEQQWAL YTDGSIRSVQ**
451 **NTNNCLTSKD HKQGSPIVLM ACSNGWASQR** WLFR**NDGSIY NLHDDMVMDV**

501 KRSDPSLKEI ILHPYHGKPN QIWLTLF

Q06076, **Abrin-d**; Protein sequence coverage: 43%

1 QDQVIKFTTE GATSQSYKQF IEALRQRLTG GLIHDIPVLP DPTTVEERNR
51 YITVELSNSE RESIEVGIDV TNAYVVAYRA GSQSYFLRDA PASASTYLFP
101 GTQRYSLRFD GSYGDLERWA HQTREEISLG LQALTHAISF LRSGASNDEE
151 KARTLIVIIQ MASEAARYRC ISNRVGVSIR TGTAFQPDPA MLSLENNWDN
201 LSGGVQQSVQ DAFPNNVILS SINRQPVVVD SLSHPTVAVL ALMLFVCNPP
251 NANQSPLLIR SIVEESKICS SRYEPTVRIG GRDGMCVDVY DDGYHNGNRI
301 IAWKCKDRLE ENQLWTLKSD LTIRSNGKCL TTEGYAPGNY VMIYDCTSAV
351 AEATYWEIWD NGTIINPKSA LVLSAESSSM GGTLTVQTNE YLMRQGWRTG
401 NNTSPFVTSI SGYSDLCMQA QGSNVWLADC DNNKKEQQWA LYTDGSIRSV
451 QNTNNCLTSK DHKQGSPIVL MACSNGWASQ RWLFKNDGSI YSLYDDMVMD
501 VKGSDPSLKQ IILWPYTGKP NQIWLTLF

Figure S7. Protein sequence coverage of proteins identified in the purified APA preparation. Approximately 85 µg of the purified APA preparation used in this work were subjected to immuno-affinity enrichment using a mixture of four mAbs, namely AP430, AP3808, AP3659 and AP476, coupled to magnetic Dynabeads, followed by protein reducing, alkylation, tryptic digest and non-targeting LC-ESI-MS/MS analysis. From top to bottom, sequences of identified *Abrus precatorius* Agglutinin (APA; UniProt Q9M6E9) and three abrin isoforms (UniProt P11140, UniProt Q06077, UniProt Q06076) are shown after MASCOT server search against a self-assembled UniProt/NCBI database containing all abrin isoforms and *Abrus precatorius* agglutinin as well as an NCBI database containing all *Abrus precatorius* proteins. Asparagine (N) highlighted in turquoise represents potential N-linked glycosylation sites. The linker peptide sequence between the two chains of each abrin isoform is underlined. Amino acids highlighted in red were experimentally identified. Amino acids in position 1–20 in APA represent the signal peptide (highlighted in grey).

References

1. Herrmann, M.S.; Behnke, W.D. A characterization of abrin A from the seeds of the *Abrus precatorius* plant. *Biochim. Biophys. Acta* **1981**, *667*, 397–410, doi:10.1016/0005-2795(81)90206-3.
2. Hegde, R.; Maiti, T.K.; Podder, S.K. Purification and characterization of three toxins and two agglutinins from Abrus precatorius seed by using lactamyl-Sepharose affinity chromatography. *Anal. Biochem.* **1991**, *194*, 101–109, doi:10.1016/0003-2697(91)90156-n.
